# *In Vitro* Study to Evaluate the Efficacy of Ultrasonicated Ethanolic Extract of *Rosmarinus officinalis* and its Chitosan-Based Nanoparticles Against *Eimeria tenella* Oocysts of Chickens

**DOI:** 10.1208/s12249-022-02445-z

**Published:** 2022-11-03

**Authors:** Shaimaa M. Kasem, Nabila M. Mira, Magdy E. Mahfouz, Ibrahim B. Helal

**Affiliations:** 1 Zoology Department, Faculty of Science, Kafrelsheikh University, Kafr ElSheikh, 33516 Egypt; 2 Zoology Department, Faculty of Science, Tanta University, EL Gharbia, 31527 Egypt

**Keywords:** anticoccidial, chitosan nanoparticles, *Eimeria tenella* oocyst, rosemary extract, sporulation

## Abstract

In this study, chitosan nanoparticles (CsNPs) were used as nanocarrier for ultrasonicated ethanolic extract of *Rosmarinus officinalis* (UEERO) as a new nanoformulation against *Eimeria tenella*. Herein, CsNPs have been synthesized by ionic gelation method at pH 3 (CsNPs3) and pH 5 (CsNPs5), followed by characterization of morphology, size, polydispersity index (PDI), surface charge, and loading efficiency of UEERO. An *in vitro* sporulation inhibition assay (10, 5, 2.5, 1.25, 0.62, 0.31, 0.15, 0.07, 0.04, 0.02, and 0.01 mg/ml normal saline solution) against *E. tenella* was conducted. Results showed that free CsNPs and UEERO-CsNPs3/5 were cubic- and spherical-shaped with positive charge and average size of ~ 150.8 nm (314.4 nm) and 151.7 nm (321.1 nm), respectively. The total loading efficiency using UV–vis spectrophotometer, was 80.05 at pH 5 and 64.39% at pH 3. The *in vitro* sporulation inhibition assay revealed that UEERO, CsNPs3/5, and UEERO-CsNPs3/5 showed a potential inhibitory effect on sporulation (%), distortion in wall (%), and sporocyst abnormality (%) in a dose-dependent manner. Accordingly, the concentration (10 mg/ml) showed the best efficacy after 24 h in UEERO, free CsNPs, and UEERO-CsNPs. Moreover, UEERO-CsNPs3 and UEERO-CsNPs5 had stopped the sporulation (%) after 72 h. Taken all together, UEERO-CsNPs3 and UEERO-CsNPs5 are best effective against *E. tenella* in a dose-dependent manner in terms of sporulation (%), distortion in wall (%), and sporocysts abnormality.

**Graphical abstract:**

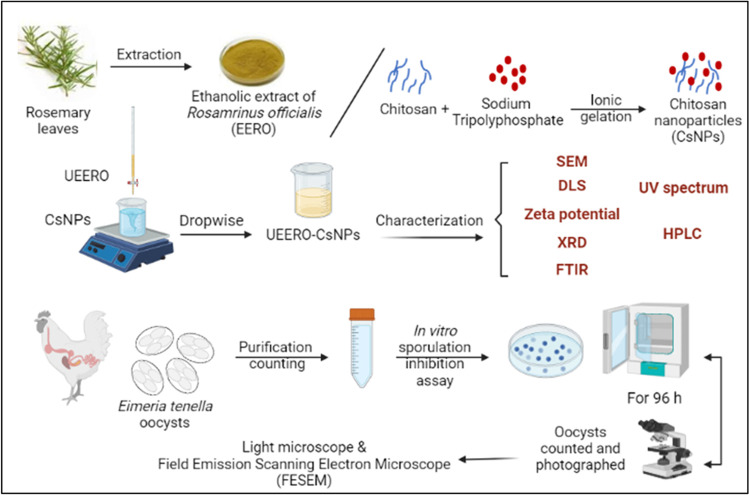

**Supplementary Information:**

The online version contains supplementary material available at 10.1208/s12249-022-02445-z.

## Introduction

Poultry coccidiosis is a severe parasitic disease caused by *Eimeria* species that are obligate intracellular Apicomplexan protozoan parasites [1]. This disease causes different clinical symptoms including diarrheal feces, poor weight gain, and high mortality [2]. Therefore, it causes huge economic losses to poultry industry [3] that reach up to USD 3 billion dollars worldwide [4, 5]. *Eimeria* parasite undergoes a direct life cycle with transmission between hosts by ingestion of a resistant infectious sporulated oocyst [6]. There are seven major *Eimeria* species that are responsible for chicken coccidiosis, including *E. tenella*, *E. necatrix*, *E. acervulina*, *E. brunetti*, *E. maxima*, *E. mitis*, and *E. Praecox* [7]. Each species has a specific site in the gastrointestinal tract [8], as well as differentiating characteristics in the appearance of macroscopic lesions, the morphology of the oocysts, the minimum sporulation time, the minimum prepatent period [9], and the size of the schizont [10, 11]. *E. tenella* is a host- and tissue-specific parasite, replicating in the epithelial cells that line the caeca of the domestic chicken [12] that causes caecal coccidiosis and is considered to be the most pathogenic species [7]. The oocyst wall is protected by a double wall of proteins and fats that give it great resistance to mechanical and chemical damage from the environment [6]. It is also resilient to proteolysis and disinfectants as well as many detergents [13, 14]. Poultry coccidiosis can be treated using synthetic anticoccidial drugs, but their continuous use can lead to development of anticoccidial drug resistance, and therefore, its use had become inefficient in controlling the disease [3, 15]. This corresponds the researchers to find alternatives to control chicken coccidiosis [16, 17]. Plant-derived compounds and their products have shown better anticoccidial effects as *Camellia sinensis* (green tea) extract, ethanolic leaf extract of *Citrus aurantium*, and *Rosmarinus officinalis* leaf extracts [1, 18, 19]. The plant rosemary (*Rosmarinus officinalis*) is an aromatic plant known for its pharmacological and therapeutic properties [20]. Its efficacy is contributed to its biologically active phytochemicals that have anti-inflammatory [21], antioxidant [22], anticancer [23], and antiparasitic [19] effects.

Nowadays, nanoparticles have been used in various domains of nanomedicine as diagnosis, bioimaging, drug delivery, and vaccine development [24]. Nanotechnology begins to create new alternative drugs for parasitic diseases [25, 26]. Jain [27] reported that forms of nanoparticles have been more efficiently than the original compound itself. Biodegradable nanoparticles represent the most important categories that have a great interest in the recent years to be applied in this field such as nanocellulose, nanochitosan, and nano-PLGA. Chitosan is a natural polysaccharide obtained mainly from the crustacean shells by the deacetylation of chitin extracted from the shells [28, 29]. It has a wide range of biological pharmacological activities such as bacteriostatic, antioxidant, immunomodulatory, and antitumor [30]. Chitosan nanoparticles (CsNPs) can be prepared by different techniques as the emulsion method, ionic gelation method, reverse micellar method, and self-assembling method [31]. Recently, CsNPs have been proven to be an effective anti-fungal, anti-bacterial and anti-protozoal agent [32]. The antiparasitic effect of CsNPs was also observed as *in vitro* and *in vivo* anti-Trypanosoma, anti-Toxoplasma, anti-Leishmania, anti-Plasmodium [33–36], and anti-cryptosporidium [32]. Additionally, in recent study reported by Elmi *et al*. [37] indicated that CsNPs inhibited protozoan growth of *Plasmodium falciparum*, *Giardia lamblia*, and *Trichomonas vaginalis in vitro*. Based on previous study, it was reported that revealed that the lowest concentration of ethanolic extract of *Rosmarinus officinalis* had a potential *in vitro* positive effect against *E. tenella* oocysts sporulation with observed changes in their morphology [19]. However, this study showed a decrease in the anticoccidial efficacy of EERO with the increase in the extract concentration and storage time that was interpreted to the agglomeration effect in solution. Herein, this study is an attempt to decrease agglomerations in the EERO extract by ultrasonication and enhance its cellular uptake *in vivo* and oocyst itself by a biocompatible nanovehicle as CsNPs [38, 39]. Therefore, the objective of this study is to evaluate the *in vitro* anticoccidial activities of ultrasonicated ethanolic extract of *Rosmarinus officinalis* (UEERO) and its chitosan-based nanoparticles (UEERO-CsNPs) on *E. tenella* oocysts of chickens.

## Materials and Methods

### Materials

Ninety to 95% deacetylation chitosan with a LMWT of about 50 kDa was obtained from Oxford, Mumbai, India. Sodium tripolyphosphate (TPP) (Mw = 367.86 g/mol) was purchased from Sigma-Aldrich. Potassium dichromate (MW = 249.19), sodium chloride (MW = 58.44), and zinc sulfate (MW = 287.56 g/mol) were obtained from Raheja Centre, Mumbai, India. Glacial acetic acid (99.5%) and absolute ethanol (99.9%) were purchased from ADWIC, Egypt. All reagents were of analytical grade and used as received. Double-distilled water was used in the study.

### Preparation of *Rosmarinus officinalis* (Rosemary) extract

Ethanolic extract of *Rosmarinus officinalis* (ERRO) was prepared by a heat reflux extraction method as described by Kasem *et al*. [19]. Briefly, the dried leaves of rosemary were ground using an electric blender to obtain fine powder. This powder was then stirred with absolute ethanol at a volume ratio of (1 powder: 5 solvent) at 50°C for at least 24 h. The resulting mixture was filtrated with Buchner funnel equipment through filter paper to remove the leaves residues. The filtrate was evaporated with high-capacity evaporator (EYELA Rotary vacuum evaporator NE-1 system) at 55°C and the final dried extract was kept in amber bottles at 4°C for further use. UEERO was prepared by sonication of the extract EERO using 40 kHZ Ultrasonic Water Bath (PT-ZPS-3A, PRISMA TECH, USA) for 15 min.

### Standardization of Rosemary Extract

#### Identification and Quantification of Polyphenolic Compounds in Rosemary Extract

The high performance liquid chromatography (HPLC) analysis was performed to identify and quantify some polyphenolic components present in EERO. HPLC analysis was carried out using an Agilent 1260 series. The separation was carried out using Eclipse C18 column (4.6 mm × 250 mm i.d., 5 μm). The mobile phase consisted of water (A) and 0.05% trifluoroacetic acid in acetonitrile (B) at a flow rate 1 ml/min. The mobile phase was programmed consecutively in a linear gradient as follows: 0 min (82% A); 0–5 min (80% A); 5–8 min (60% A); 8–12 min (60% A); 12–15 min (82% A); 15–16 min (82% A); and 16–20 (82%A). The multi-wavelength detector was monitored at 280 nm. The injection volume was 5 μl for each of the sample solutions. The column temperature was maintained at 40°C.

### EDX

Energy-dispersive X-ray spectroscopy (EDX) analysis was performed to screen the elemental composition of EERO using the silicon–drift EDS detector (energy resolution about 129 eV or better) with the analysis condition of WD 10 mm and voltage 20 kV.

### Synthesis of CsNPs

Chitosan nanoparticles were synthesized using ionic gelation method [40, 41]. Chitosan (1 mg/ml) was prepared in 1% of glacial acetic acid with a continuous stirring overnight and filtered until became clear yellowish solution. Chitosan nanoparticles (CsNPs) were formed after drop wise addition of 1 mg/ml double distilled H_2_O of sodium tripolyphosphate (TPP) into chitosan solution (1 mg/ml) with a volume ratio (1:5, TPP:CS, v/v) under magnetic stirring of 500 rpm at room temperature for 1 h. The synthesized NPs were synthesized at different pH 3 and 5.

### Synthesis of UEERO-CsNPs

**Scheme 1:**
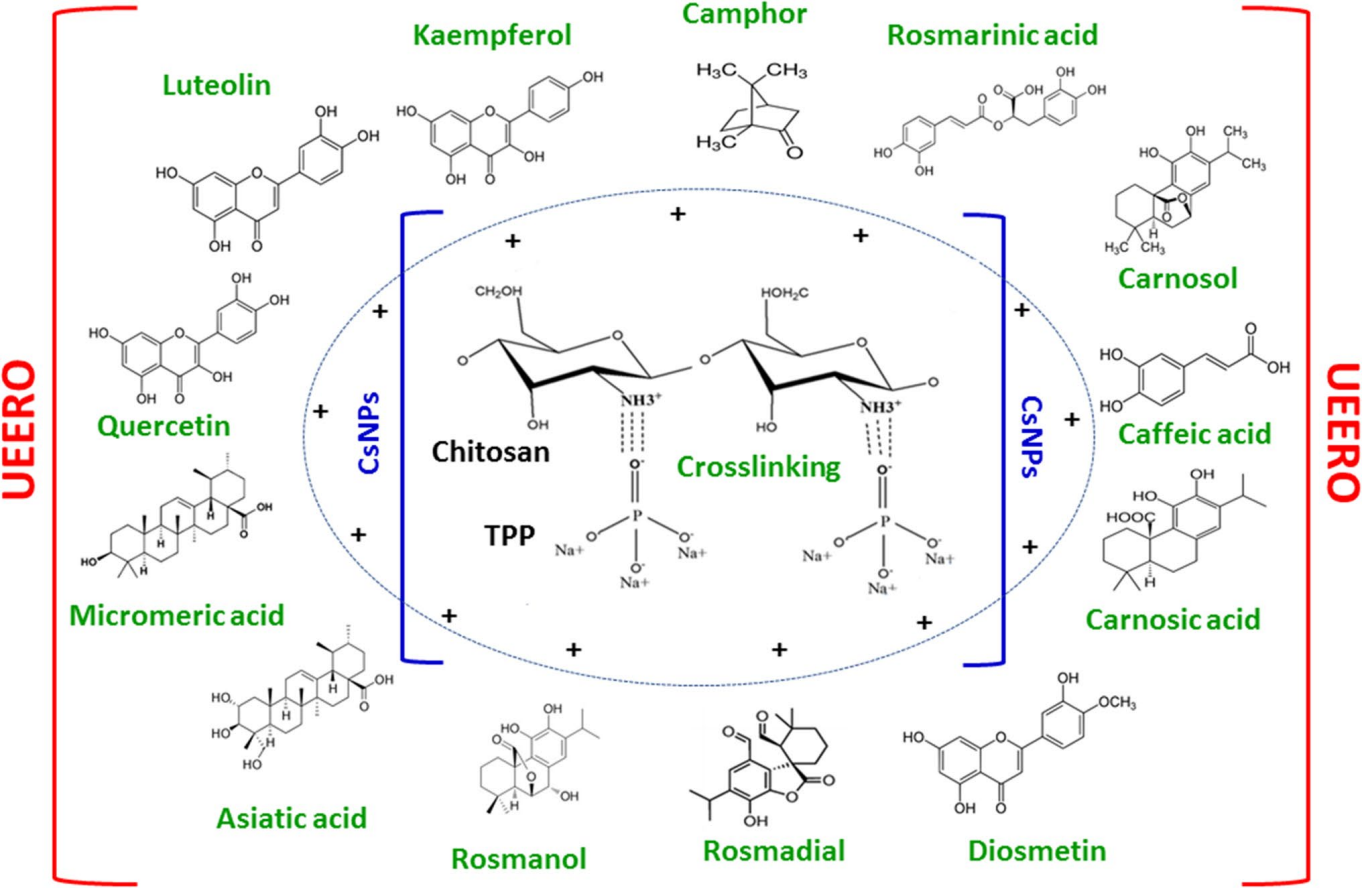
The mechanism of Cs–TPP crosslinking and interaction with phenolic components of UEERO during the synthesis process

Ultrasonicated ethanolic extract of *Rosmarinus officinalis* (UEERO) (1 mg/ml) was dispersed in absolute ethanol with 15 min of sonication. UEERO and TPP solutions (1 mg/ml) were mixed at (5:1, v/v) for 1 h at 500 rpm magnetic stirring. UEERO-TPP solution was added drop by drop to Cs solution (1 mg/ml) using UEERO and Cs solutions at the same volume (1:1, v/v) at 500 rpm and pH 3 and 5 for 1 h as shown in Scheme 1.

### Characterization Techniques

Morphology of the synthesized NPs was investigated using scanning electron microscopy (SEM), JEOL SEM, JSM-IT100. The samples were diluted in a ratio of 1:10 and air dried. Then, the samples were sputter coated with gold for 2 min.

The particle size and zeta potential were measured at samples diluted in a ratio of 1:10 at 25°C using Brookhaven Nano-ZS and Malvern zeta size, respectively. The powder X-ray diffraction (XRD) patterns of the samples were described on a Shimadzu – XRD 6000, X-ray diffractometer with Cu Kα radiation (λ = 1.5418 Å). The Fourier Transform Infrared (FTIR) analysis was performed using JASCO, FTIR- 6800 Spectrometer. The UV–Vis absorption spectra were carried out by spectrophotometer (JASCO-V-730) within the wavelength range of 200–1100 nm.

### Total LE

The whole free UEERO compounds were separated in supernatant from UEERO-CsNPs solution following cooling centrifugation at 13,000 rpm at 10°C for 1 h to separate free components in the supernatant and the bounded in the pellet. The absorbance can be calculated by UV–vis spectrophotometer at a wavelength of λmax = 280 nm, and so, the concentrations were detected from the standard curve of the known concentrations from the extract. Finally, the total loading efficiency (LE) of UEERO on CsNPs was calculated according to Espinoza *et al*. [42] by the following equation:

1 $$\mathrm{Loading}\;\mathrm{effeciency}\;\left(\mathrm{LE}\right)\%=\frac{\mathrm{Total}\;\mathrm{UEERO}\left(\mathrm{CT}\right)-\mathrm{Free}\;\mathrm{UEERO}\; {\texttt{"}} \mathrm{supernatant} {\texttt{"}} \;\left({\mathrm C}_0\right)}{\mathrm{Total}\;\mathrm{UEERO}\;\left(\mathrm{CT}\right)}\times100$$

where LE, loading efficiency; CT, total concentration of UEERO; and C_0_, free UEERO concentration.

### HPLC

The loading efficiency of caffeic and rosmarinic acids as the most potent antioxidant compounds involved in UEERO extract was also calculated using HPLC analysis. HPLC analysis of UEERO, UEERO-CsNPs3, and UEERO-CsNPs5 was carried out using an Agilent 1260 series. The separation was carried out using Eclipse C18 column (4.6 mm × 250 mm i.d., 5 μm). The mobile phase consisted of water (A) and 0.05% trifluoroacetic acid in acetonitrile (B) at a flow rate 1 ml/min. The mobile phase was programmed consecutively in a linear gradient as follows: 0 min (82% A); 0–5 min (80% A); 5–8 min (60% A); 8–12 min (60% A); 12–15 min (82% A); 15–16 min (82% A); and 16–20 (82%A). The multi-wavelength detector was monitored at 280 nm. The injection volume was 5 μl for each of the sample solutions. The column temperature was maintained at 40°C. Finally, the LE of caffeic and rosmarinic acids was determined as described by Khursheed *et al*. [43] using the following equation:

2 $$\mathrm{Loading}\;\mathrm{effeciency}\;\left(\mathrm{LE}\right)\%=\frac{\mathrm{Total}\;\mathrm{UEERO}\;\mathrm{loaded}\;\mathrm{in}\;\mathrm{NPs}}{\mathrm{Total}\;\mathrm{UEERO}\;\mathrm{input}}\times100$$

### Collection and Sporulation of *E. tenella* Oocysts

A strain of unsporulated *E. tenella* oocysts was initially isolated by scrapping from the ceca of naturally infected coccidian chicks, preserved in 2.5% potassium dichromate (K_2_Cr_2_O_7_) solution [44] at 2–5°C for storage until use which prevents bacterial, fungal degradation and petrifaction of oocysts. Identification of the species was based on the morphological characteristics described by Thienpont *et al*. [45], as well as the site of lesions [46]. The collected oocysts were sporulated in 2.5% potassium dichromate solution at 25–29°C for 48 h and 60–80% relative humidity [19, 44].

### Counting *E. tenella* Oocysts

Oocysts per gram feces (OPG) were counted according to Long and Rowell [47] and Long *et al*. [48] by using McMaster counting chamber technique.

### Propagation, Isolation, and Purification of *E. tenella* Oocysts

Twenty-one-day-old white healthy broiler chicks were purchased from a local hatchery from Kafr-Elsheikh City, Egypt. Chicks were reared in wire-floored cages with a double tray and wood shavings as bedding material to catch fecal material. Tap water and commercial food of ordinary ration without any anticoccidial drugs and antibiotics were used. Animals were acclimatized and kept in an animal facility room with regulated temperature (25–29°C) and light/dark cycle (18/6 h). At day 14, each chick was orally inoculated (intra-crop) with 1 ml of inoculum containing 4 × 10^4^ viable *E. tenella* sporulated oocysts to induce infection [19]. Directly before inoculation, fecal samples from the chicks were tested and shown to be free from *Eimeria* oocysts. Six days after infection, the suspected infected chicks were humanly sacrificed and ceca were separated and cut longitudinally with scissors. The cecal contents were collected by scrapping from the lesions in normal saline solution; 0.9% sodium chloride (NaCl), well homogenized sieved through a fine wire mesh to discard the debris and left to be concentrated by precipitation for 20 min. The filtrate containing umsporulated oocysts then collected, re-suspended in 2.5% potassium dichromate solution and stored at 2–5°C until use. Also, unsporulated oocysts were collected and purified from the fecal matter by concentration flotation technique using zinc sulfate flotation technique acco[rding to the method of Levine [49], preserved in 2.5% potassium dichromate solution and stored at 2–5°C until use. Photomicrographs of unsporulated and sporulated oocysts were taken with a LEICA ICC50 HD microscope camera (Germany) using LAS EZ imaging software (version 2.1.0) and the size of oocysts was recorded.

### *In Vitro* Anticoccidial Test

An *in vitro* anticoccidial test was conducted using sporulation inhibition assay to estimate the effects of UERRO, free CsNPs3, free CsNPs5, UERRO-CsNPs3, and UERRO-CsNPs5 against the sporulation of *E. tenella* oocysts. For this purpose, 10 mg/ml normal saline solution of each the tested materials were prepared as a stock solution. Ten serial dilutions (5, 2.5, 1.25, 0.62, 0.31, 0.15, 0.07, 0.04, 0.02, and 0.01 mg/ml normal saline solution) were prepared from the stock solutions in Petri dishes [19]. Unsporulated oocysts (2 × 10^4^) were added to each Petri dish. Two Petri dishes containing normal saline solution were served as control groups. Triplicates were made from each concentration of the tested materials. All Petri dishes were partially covered, to allow the passage of oxygen and incubated at 25–29°C for 96 h and 60–80% humidity. The contents of the Petri dishes were stirred off and on to ensure the oxygenation. After 24, 48, 72, and 96 h, the sporulated and unsporulated oocysts were observed and counted under inverted light microscope at 40 × . The sporulation percentage (%) was estimated by counting the number of sporulated ones in a total of 100 oocysts. Any deformations observed in sporocysts and oocysts wall were recorded at 400 × and photomicrographs were taken with a LEICA ICC50 HD microscope camera (Germany) using LAS EZ imaging software (version 2.1.0).

### FESEM of *E. tenella* Oocysts

To verify changes occurred during this *in vitro* study due to the usage of different tested materials including UEERO, free CsNPs3 and 5 as well as UEERO-CsNPs3 and 5, the surface of *E. tenella* oocysts was examined by field emission scanning electron microscopy (FESEM). However, because the concentration 10 mg/ml of all these tested materials had the lowest percentage of sporulation in comparison to the other concentrations, only oocysts treated with the 10 mg/ml was further examined by FESEM. Oocysts were pelleted by centrifugation at 2000–3000 rpm for 10 min and fixed with 2.5% buffered glutaraldehyde for 24–48 h. Then, the samples were dehydrated into ascending concentrations of ethanol (50, 60, 70, 80, 90, 95, 100, and 100%) for 5–10 for each. Finally, the dehydrated samples were dried by critical point drier. The morphology of oocysts was observed and photographs were taken using FESEM, Quattro S FEG SEM – Thermo Fisher, NL operated between 15 and 20 keV.

### Statistical Analysis

Data were analyzed using Statistical Package for Social Science (SPSS, version 20). One-way analysis of variance (ANOVA) was used. Tukey test was applied in order to determine the statistical differences between means. For values not normally distributed, the non-parametric analysis of Mann–Whitney *U* test was employed. The results are presented as (mean values ± standard deviation) and considered statistically significant when probability values (*P* values) were less than 0.05 (*P* ≤ 0.05).

## Results

### Standardization of Rosemary Extract

The HPLC polyphenolic compounds profiles for EERO are shown in supplementary material Table SI and the representative HPLC chromatograms of the multi-standards and EERO are also shown in Fig. S1. In the present study, different groups of 13 polyphenolic compounds have been recognized in EERO using HPLC analysis including phenolic acids, phenolic aldehydes and flavonoids. About 9 phenolic compounds including 8 phenolic acids (ferulic acid, 1009.25 µg/g; gallic acid, 1004.16; ellagic acid, 739.12 µg/g; syringic acid, 546.17 µg/g; chlorogenic acid, 399.66 µg/g; methyl gallate, 150.54 µg/g; coumaric acid, 72.51 µg/g; and cinnamic acid, 40.81 µg/g) and one phenolic aldehyde (vanillin; 72.23 µg/g) were recognized in EERO. In addition, a total of 4 flavonoid compounds belonging to the two subclasses; flavonols (kaempferol, 259.04 µg/g, and quercetin, 179.23 µg/g) and flavanones (hesperetin, 135.50 µg/g, and naringenin, 27.65 µg/g) were identified. Moreover, catechin, pyro catechol, and rutin were not appeared in EERO.

Figure S1 shows the EDX analysis that reveals that only the characteristic peaks of C (0.227 keV), O (0.525 keV), and K (3.312 keV) with a mass fraction (wt.%) of about 73.46, 26.35, and 0.19%, respectively. Also, atomic fractions of C, O, and K are about 78.73, 21.20, and 0.06%, respectively.

## Characterization of CsNPs and UEERO-CsNPs

**Fig. 1:**
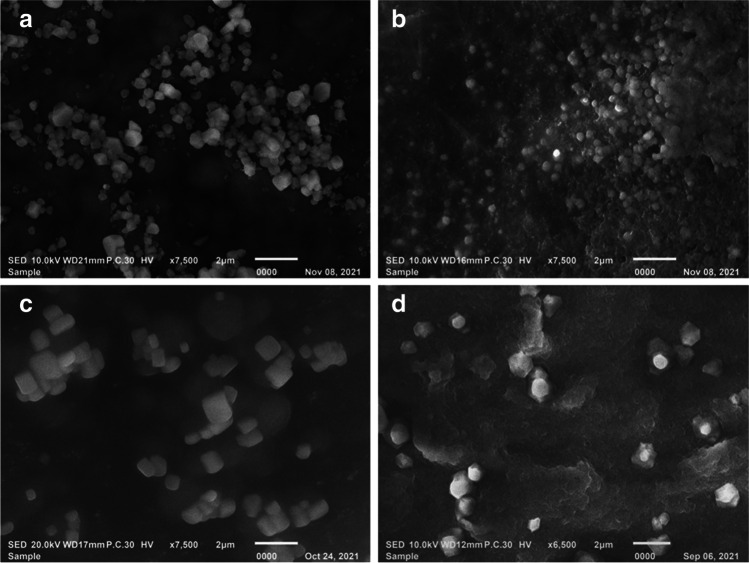
SEM micrographs of **a** Free CsNPs3, **b** Free CsNPs5, **c** UEERO-CsNPs3, and **d** UEERO-CsNPs5. UEERO; ultrasonicated ethanolic extract of *Rosmarinus officinalis* free CsNPs3; free chitosan nanoparticles at pH 3 free CsNPs5; free chitosan nanoparticles at pH 5 UEERO-CsNPs3; ultrasonicated ethanolic extract of *Rosmarinus officinalis*-chitosan based nanoparticles at pH 3 UEERO-CsNPs5; ultrasonicated ethanolic extract of *Rosmarinus officinalis*-chitosan based nanoparticles at pH 5. Scale bar = 2 µm

Figure 1 shows SEM micrographs of free and loaded CsNPs that showed the cubic and spherical shape of the synthesized NPs at pH 3 and pH 5, respectively.

Figure S2 indicates that the average hydrodynamic size of CsNPs3 and CsNPs5 are about 150.8 and 151.3 nm, respectively. Accordingly, the size of UEERO-CsNPs3 (314.8 nm) exhibited a small decrease than UEERO-CsNPs5 (321.1 nm) but less than the size of free UEERO that was 2808.4 nm (≈ 3 µm). Also, the positive zeta potential of CsNPs decreased from + 38.83 mV at pH 5 to + 20.47 mV at pH 3. The decrease in positive charge of loaded CsNPs is an indication of the successful loading of negatively charged EERO particles, especially at pH 5 than pH 3. Otherwise, all synthesized samples showed an acceptable value of PDI (≤ 0.3) that indicates the good dispersion and homogeneity of samples [50].

Zeta potential of NPs have a positive charge that exhibited a decrease from + 38.83 mV at pH 5 to + 20.47 mV at pH 3 due to the increase of cross linked phosphoric and OH- groups of TPP that causing decrease of the number of the free positive amino groups of Cs.

**Fig. 2:**
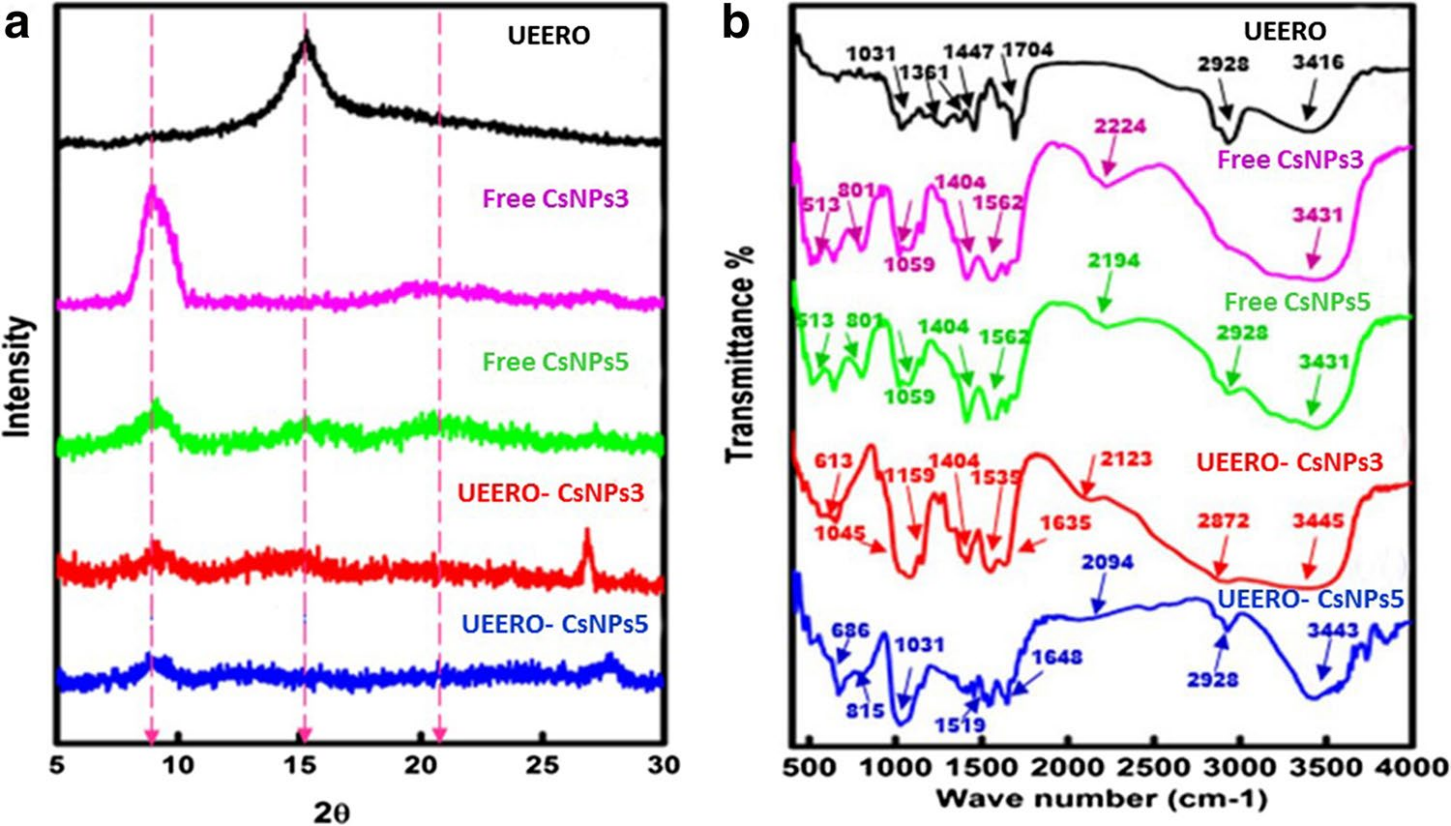
XRD patterns **a**, and FTIR spectra **b**. UEERO; ultrasonicated ethanolic extract of *Rosmarinus officinalis* free CsNPs3; free chitosan nanoparticles at pH 3 free CsNPs5; free chitosan nanoparticles at pH 5 UEERO-CsNPs3; ultrasonicated ethanolic extract of *Rosmarinus officinalis*-chitosan based nanoparticles at pH 3 UEERO-CsNPs5; ultrasonicated ethanolic extract of *Rosmarinus officinalis*-chitosan based nanoparticles at pH 5

Figure 2a shows XRD patterns of free UEERO, CsNPs and UEER0-CsNPs. Free CsNPs exhibited a crystalline peak at 2ϴ = 9.08° and another amorphous peak at 2ϴ = 20.75° [51] The sharp peak showed a decrease in intensity in case of free CsNPs5 due to the semi-crystalline nature of the synthesized NPs at pH 5. Free UEERO showed a strong sharp peak at 2ϴ = 15.26° that is expected to be affected after interaction with CsNPs as a result of deformation of crystal structure and encapsulation inside the NPs.

Figure 2b shows FTIR spectra of free UEERO, free CsNPs and UEERO-CsNPs. Free CsNPs3 and 5 showed a band at 1059 cm^−1^ is related to C–C stretching. The weak band observed at 1404 cm^−1^ is due to bending vibration of CH_2_. The bands at 1562 cm^−1^ and from 2194 to 2928 cm^−1^ are attributed to N–H bending of primary amine group and stretching vibration of C-H bonds on CH_2_, respectively. The broad band at 3430 cm^−1^, corresponds to stretching vibrations of axial OH and -NH groups. The bands appeared at the finger print region from 513 to 801 cm^−1^ is due to the cross linkage between NH_2_ groups of CsNPs and phosphoric groups of TPP [52].

UEERO exhibited a weak band at 1031 cm^−1^ corresponds C-O bond asymmetric stretching. The band observed at 1261 cm^−1^ is attributed to ether function from the epoxy ring of 1,8-cineole. The bands from 1361 to 1447 cm^−1^ and 2928 cm^−1^ contribute to C-H stretching bands on CH_2_. The band at 1704 cm^−1^ is due to camphor keto group. The broad band at 3416 cm^−1^, corresponds to stretching vibrations of axial OH and -NH groups [53]. Results revealed a shift in the band of camphor keto group and the epoxy ring of 1,8-cineole as well as the increase in C-O stretching band that ensured the good interaction of UEERO and CsNPs.

The total loading efficiency (%) measured using UV–vis spectrophotometer, was 80.05 at pH 5 and 64.39% at pH 3 (Table SII). Additionally, Figure S3 indicates HPLC results showed that in free UEERO, the characteristic peaks of caffeic and rosmarinic acids were observed at retention time of 3.38 and 11.12 min, respectively. In UEERO-CsNPs3, the characteristic peaks of caffeic and rosmarinic acids were observed at retention time of 3.41 and 11.22 min, respectively, while in UEERO-CsNPs5 were observed at retention time of 3.80 and 11.32 min, respectively (Fig. 4b, c). Consequently, the LE of both caffeic and rosmarinic acids as the most potent antioxidants involved in UEERO particles [54], aid in the suspected overall destructive effect of UEERO against coccidial oocysts, increased in case of UEERO-CsNPs3 (Table SII).

## Oocysts of *E. tenella*

**Fig. 3:**
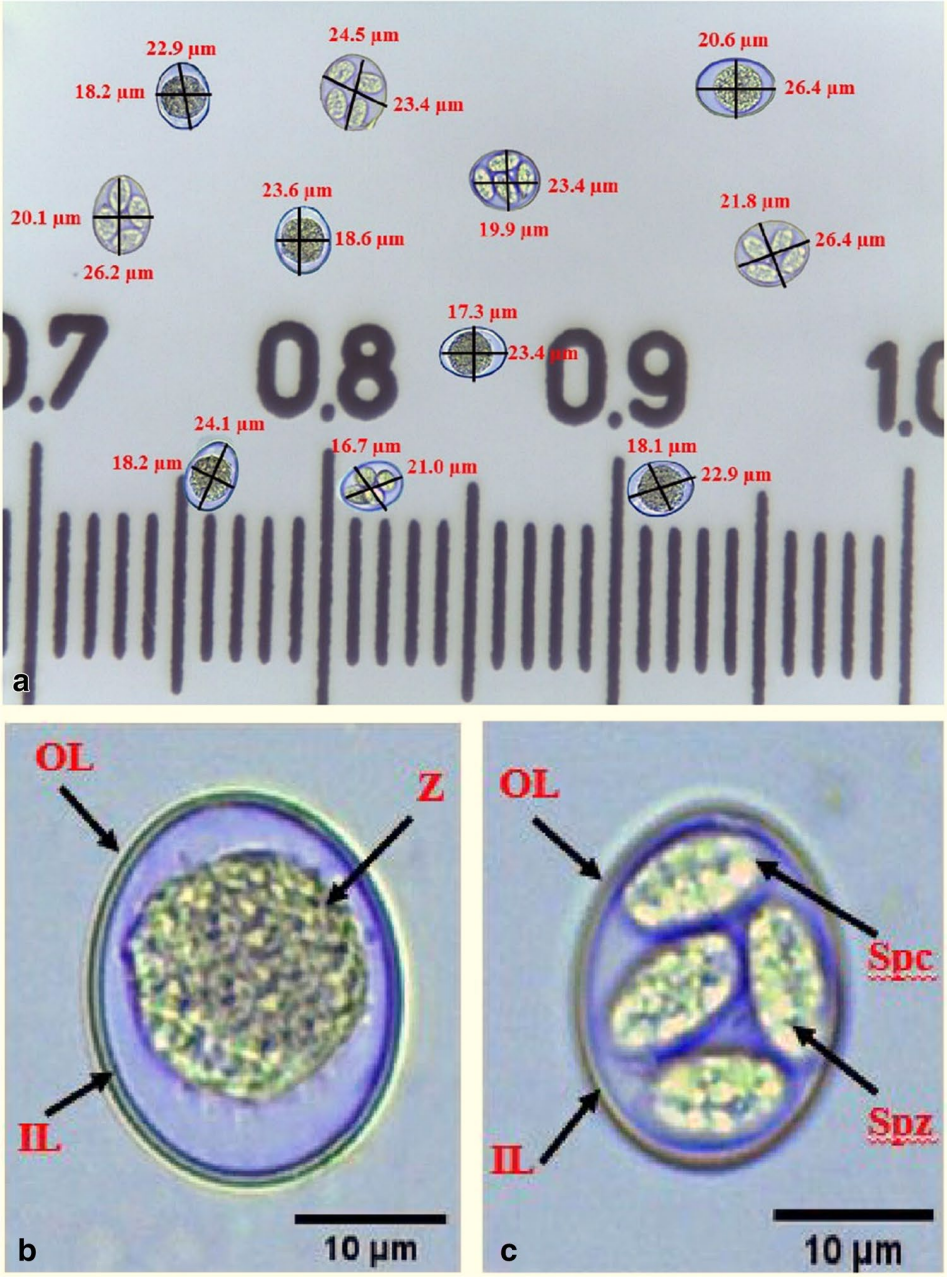
Oocysts of *E. tenella*. **a** Unsporulated and sporulated oocysts of *E. tenella*, **b** Enlarged unsporulated *E. tenella* oocyst with an inside zygote (Z) and two layered oocyst wall (outer layer; OL and inner layer; IL), and **c** Enlarged sporulated *E. tenella* oocyst surrounded by two layered oocyst wall (outer layer; OL and inner layer; IL). with 4 sporocysts (Spc) each has 2 sporozoites inside (Spz) scale bar: 10 µm

The identification of *Eimeria* species in the present study was based on the pathological sites in the host, shape and measurements of oocysts. Unsporulated oocysts of *E. tenella* in this study had showed ovoid shape with an inside zygote and surrounded by two layered oocyst wall (outer and inner) (Fig. 3b), while sporulated oocysts appeared ovoid and surrounded by two layered oocyst wall (outer and inner) with 4 sporocysts, each had 2 sporozoites inside (Fig. 3c). Additionally, oocysts of *E. tenella* of this study had an average size of 22.62 ± 1.35 µm in length, 18.81 ± 1.58 µm in width and had a shape index of 1.21 ± 0.11.

## *In Vitro* Sporulation Inhibition Assay Results

Untreated oocysts from control incubations of this study showed that sporulation percentage was 80.33% ± 1.53, 87.67% ± 2.52, 93.00% ± 2.65 and 98.00% ± 1.00 after 24, 48, 72 and 96 h respectively with no remarkable changes on shape and morphology of unsporulated (Fig. 3b) and sporulated oocysts (Fig. 3c).

## Effect of Ultrasonicated Ethanolic Extract of *Rosmarinus officinalis* (UERRO) on *E. tenella* Oocysts

**Fig. 4:**
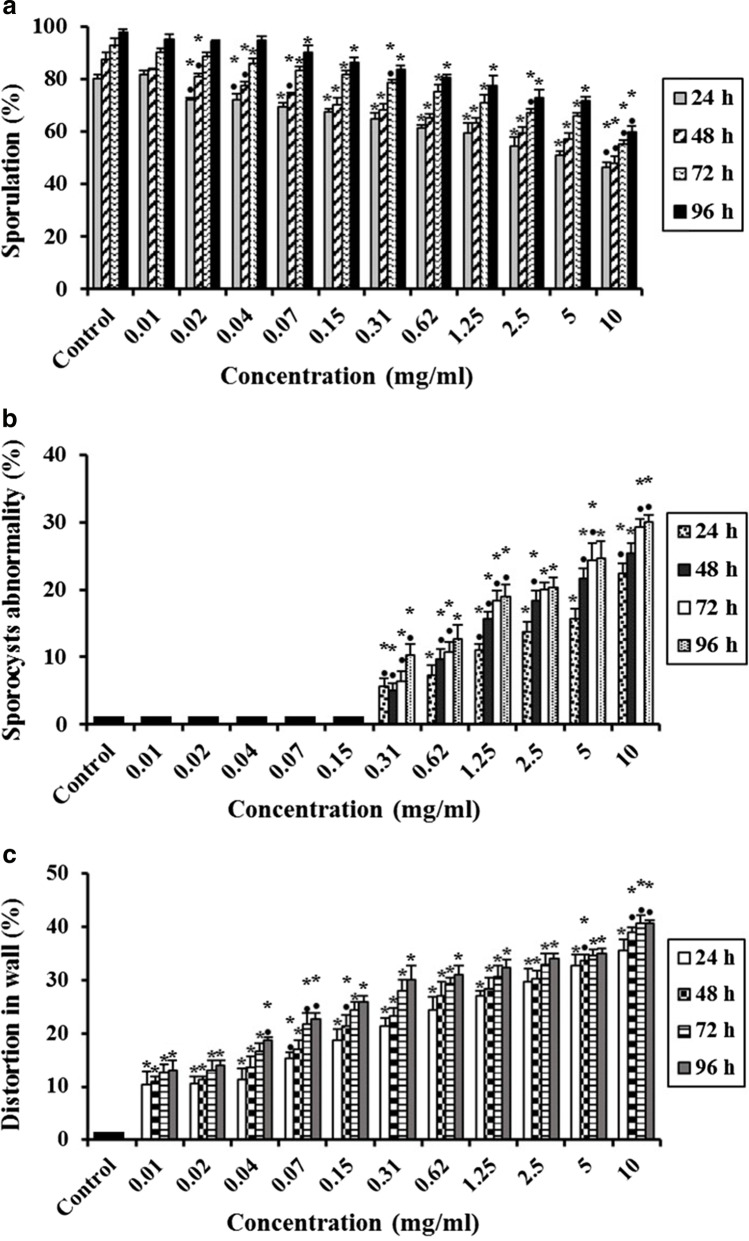
Effect of various concentrations of ultrasonicated ethanolic extract of *Rosmarinus officinalis* (UERRO) on *E. tenella* oocysts. **a** Oocysts sporulation (%), **b** Sporocysts abnormality (%), and **c** Distortion in wall (%). Data are presented as means ± standard deviation. ^*^Significant (P ≤ 0.05) when compared to control group. ^•^Significant (P ≤ 0.05) when compared with the previous lower concentration

In the present study, different concentrations of ultrasonicated UERRO (10, 5, 2.5, 1.25, 0.62, 0.31, 0.15, 0.07, 0.04, 0.02 and 0.01 mg/ml) showed significant decreases (P ≤ 0.05) in the sporulation percentage after 24, 48, 72 and 96 h as compared to the control except 0.04 mg/ml after 96 h, 0.02 mg/ml after 72 and 96 h as well as 0.01 mg/ml after 24, 48, 72 and 96 h showed non-significant changes compared to the control. As the concentration of UERRO decreased, the sporulation percentage increased. The highest concentration (10 mg/ml) of UERRO showed the lowest sporulation (%) as 46.33 ± 2.08%, 48.00 ± 2.65%, 55.33 ± 1.53% and 60.00 ± 2.00% after 24, 48, 72 and 96 h, respectively. Whereas, the lowest concentration (0.01 mg/ml) of UERRO showed the highest sporulation (%) as 81.67 ± 1.53%, 83.67 ± 0.58%, 90.33 ± 1.53% and 95.00 ± 2.00% after 24, 48, 72 and 96 h, respectively (Fig. 4a). This corresponds to that the highest concentration (10 mg/ml) revealed the highest sporulation inhibition (%) after 24 h compared to the other concentrations. Additionally, the concentrations (10, 5, 2.5, 1.25, 0.62 and 0.31 mg/ml) of UERRO had a remarkable negative effect on oocysts shape and morphology with significant (P ≤ 0.05) abnormalities in sporocysts (%) of sporulated oocysts in a dose dependent manner comparing to the control from 24 to 96 h (Fig. 4b). The highest concentration (10 mg/ml) showed the highest percentage in sporocysts abnormalities by 22.33 ± 1.53%, 25.33 ± 1.53%, 29.33 ± 1.15% and 30.00 ± 1.00% after 24, 48, 72 and 96 h, respectively (Fig. 4b). Moreover, all the tested concentrations (10, 5, 2.5, 1.25, 0.62, 0.31, 0.15, 0.07, 0.04, 0.02 and 0.01 mg/ml) exhibited significant (P ≤ 0.05) distortion percentage in oocysts wall in a dose dependent manner as related to the control (Fig. 4c). The highest concentration (10 mg/ml) revealed the highest distortion (%) in oocyst wall by 35.67 ± 2.08%, 39.00 ± 1.00%, 40.67 ± 1.53% and 40.67 ± 0.58% after 24, 48, 72 and 96 h, respectively, while the lowest concentration (0.01 mg/ml) exhibited the lowest distortion (%) in oocyst wall by 10.33 ± 2.52%, 11.00 ± 1.00%, 12.67 ± 1.53% and 13.00 ± 2.00% after 24, 48, 72 and 96 h, respectively (Fig. 4c).

## Effect of Free Chitosan Nanoparticles (CsNPs) on *E. tenella* Oocysts

**Fig. 5:**
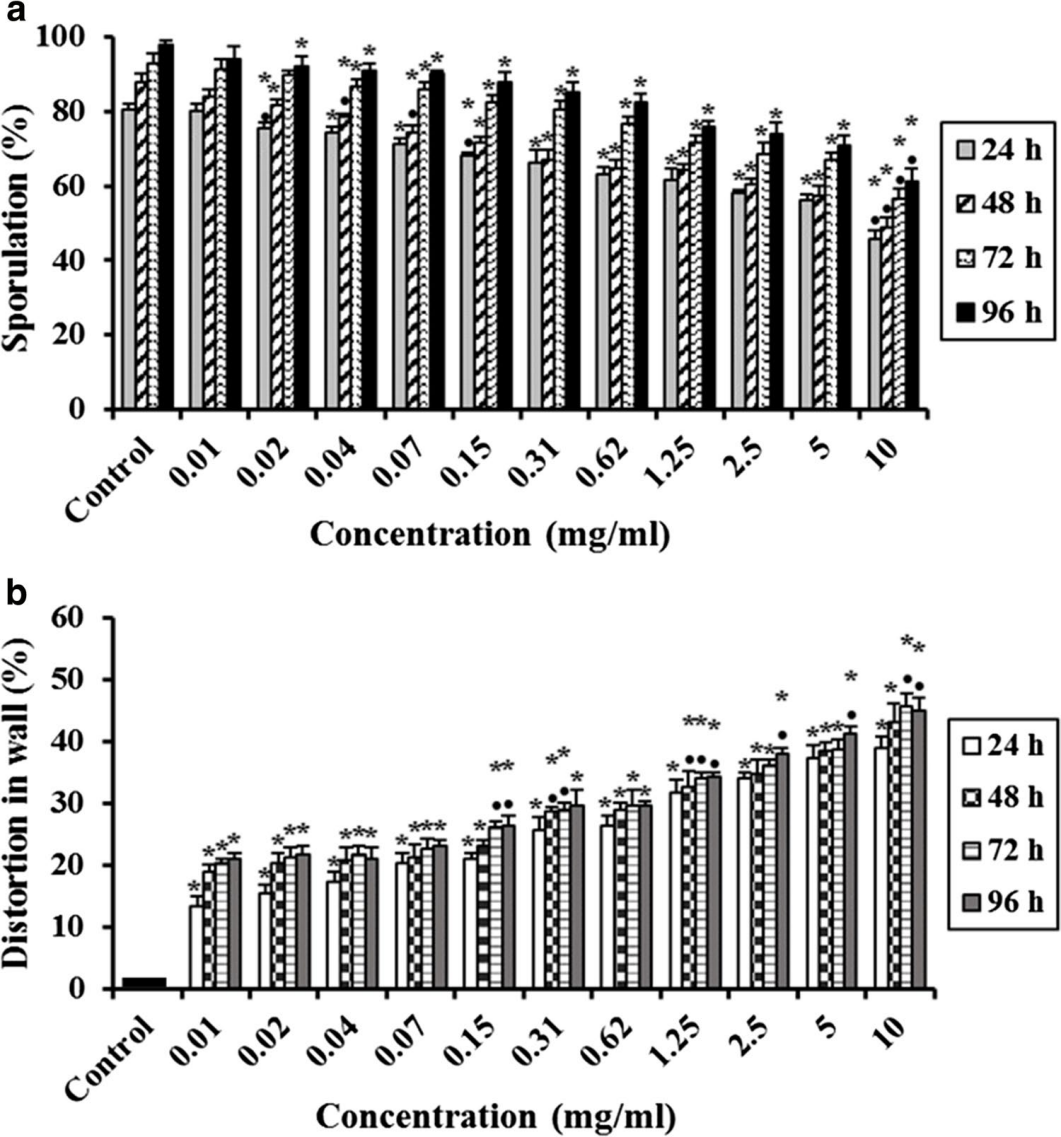
Effect of various concentrations of free chitosan nanoparticles at pH 3 (free CsNPs3) on *E. tenella* oocysts. **a** Oocysts sporulation (%), and **b** Distortion in wall (%). Data are presented as means ± standard deviation. ^*^Significant (P ≤ 0.05) when compared to control group. ^•^Significant (P ≤ 0.05) when compared with the previous lower concentration

**Fig. 6:**
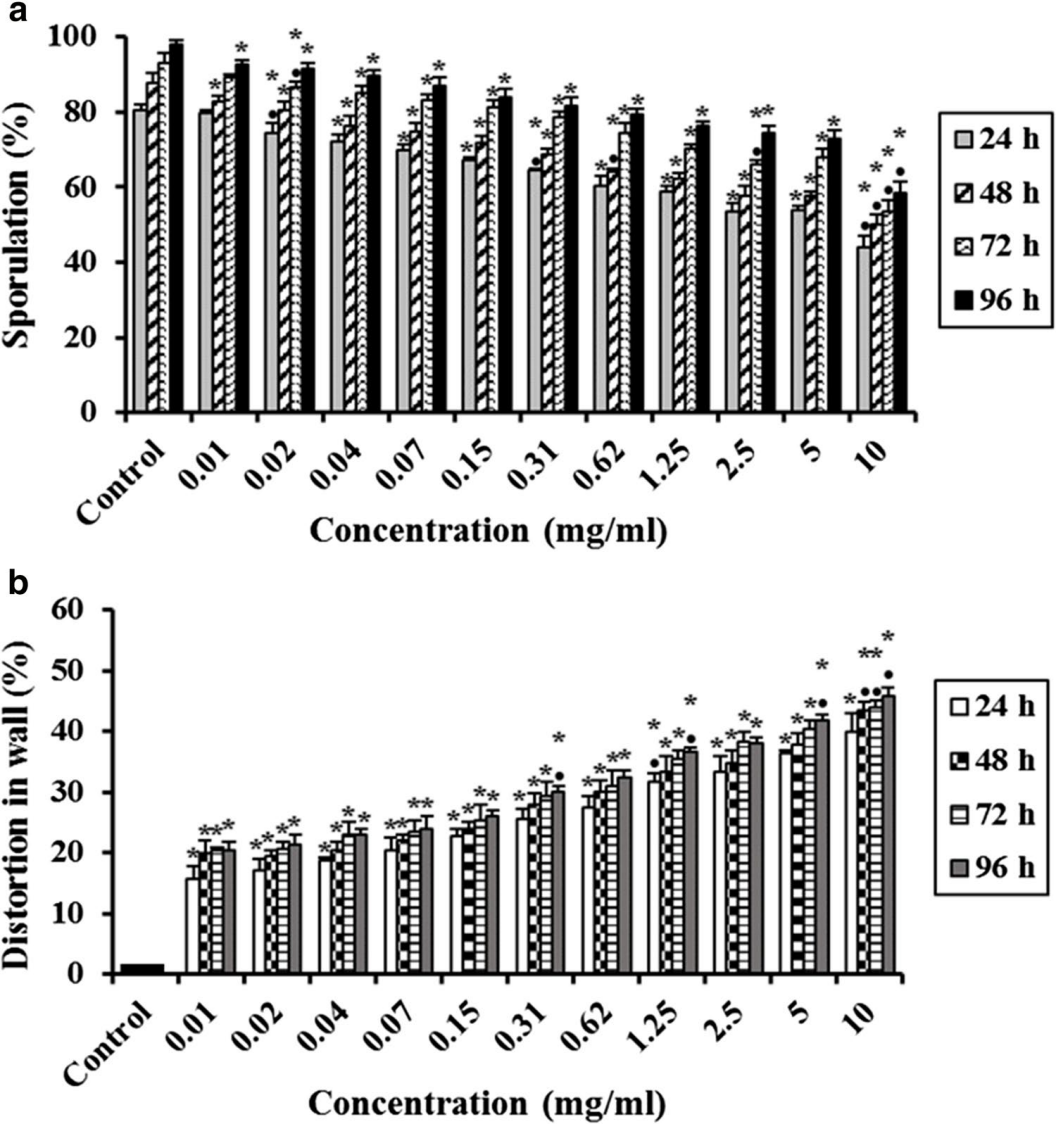
Effect of various concentrations of free chitosan nanoparticles at pH 5 (free CsNPs5) on *E. tenella* oocysts. **a** Oocysts sporulation (%), and **b** Distortion in wall (%). Data are presented as means ± standard deviation. ^*^Significant (P ≤ 0.05) when compared to control group. ^•^Significant (P ≤ 0.05) when compared with the previous lower concentration

The efficacy of free CsNPs at pH 3 &5 on *E. tenella* oocysts sporulation (%) in the herein study is shown in Figs. 5a and 6a, respectively. All the tested concentrations exhibited significant decreases (P ≤ 0.05) in the sporulation (%) at all times from 24 to 96 h except 0.02 mg/ml at 72 h and 0.01 mg/ml from 24 to 96 h in case of free CsNPs3 as well as 0.01 mg/ml at 24 h in case of free CsNPs5 in comparing to the control. As the concentration of free CsNPs decreased, the sporulation percentage increased. The lowest sporulation (%) was observed at 10 mg/ml and the highest sporulation (%) was seen in the lowest concentration (0.01 mg/ml) in either free CsNPs3 or 5 in comparison to the control group (Figs. 5a and 6a).

Concerning free CsNPs3, the lowest sporulation (%) was 45.67 ± 2.31%, 49.00 ± 2.65%, 56.67 ± 2.52% and 61.33 ± 3.21% at 10 mg/ml causing significant (P ≤ 0.05) distortion in the oocyst wall (%) by 39.00 ± 1.73%, 43.00 ± 3.00%, 45.67 ± 2.08% and 45.00 ± 2.00% after 24, 48, 72 and 96 h, respectively (Fig. 5a and b), while the lowest concentration (0.01 mg/ml) showed non-significant changes in the sporulation percentage comparing to the control but it caused significant (P ≤ 0.05) distortion in the oocyst wall (%) by 13.33 ± 1.53%, 19.00 ± 1.00%, 20.33 ± 0.58%% and 21.00 ± 1.00% after 24, 48, 72 and 96 h, respectively (Fig. 5a and b). On the other hand, using free CsNPs5, the lowest sporulation (%) was 44.00 ± 3.00%, 50.00 ± 2.65%, 53.33 ± 3.21% and 58.33 ± 3.06% after 24, 48, 72 and 96 h, respectively was recorded at 10 mg/ml showing significant (P ≤ 0.05) distortion in the oocyst wall (%) by 40.00 ± 3.00%, 43.33 ± 1.53%, 44.00 ± 1.00% and 45.67 ± 1.53% after 24, 48, 72 and 96 h, respectively comparing to the control (Fig. 8a and b), while 0.01 mg/ml showed the highest sporulation (%); 79.67 ± 0.58%, 82.67 ± 1.53%, 89.33 ± 0.58% and 92.67 ± 1.15% with significant (P ≤ 0.05) distortion in the oocyst wall (%) by 15.67 ± 2.08%, 20.00 ± 2.00%, 20.33 ± 0.58% and 20.33 ± 1.53% after 24, 48, 72 and 96 h, respectively comparing to the control (Fig. 6a and b).

## Effect of Ultrasonicated Ethanolic Extract of *Rosmarinus officinalis*-Chitosan Based Nanoparticles (UERRO-CsNPs) on* E. tenella* Oocysts

**Fig. 7:**
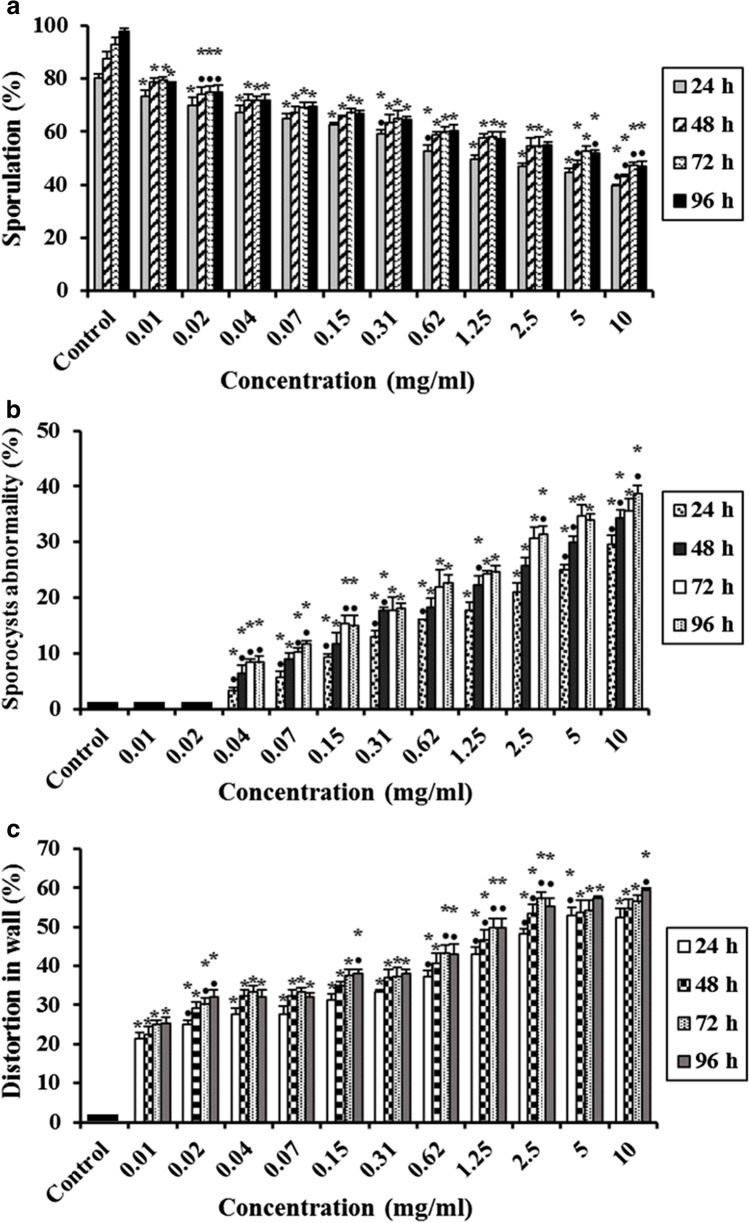
Effect of various concentrations of ultrasonicated ethanolic extract of *Rosmarinus officinalis*-chitosan based nanoparticles at pH 3 (UERRO-CsNPs3) on *E. tenella* oocysts. **a** Oocysts sporulation (%), **b** Sporocysts abnormality (%), and **c** Distortion in wall (%). Data are presented as means ± standard deviation. ^*^Significant (P ≤ 0.05) when compared to control group. ^•^Significant (P ≤ 0.05) when compared with the previous lower concentration

**Fig. 8:**
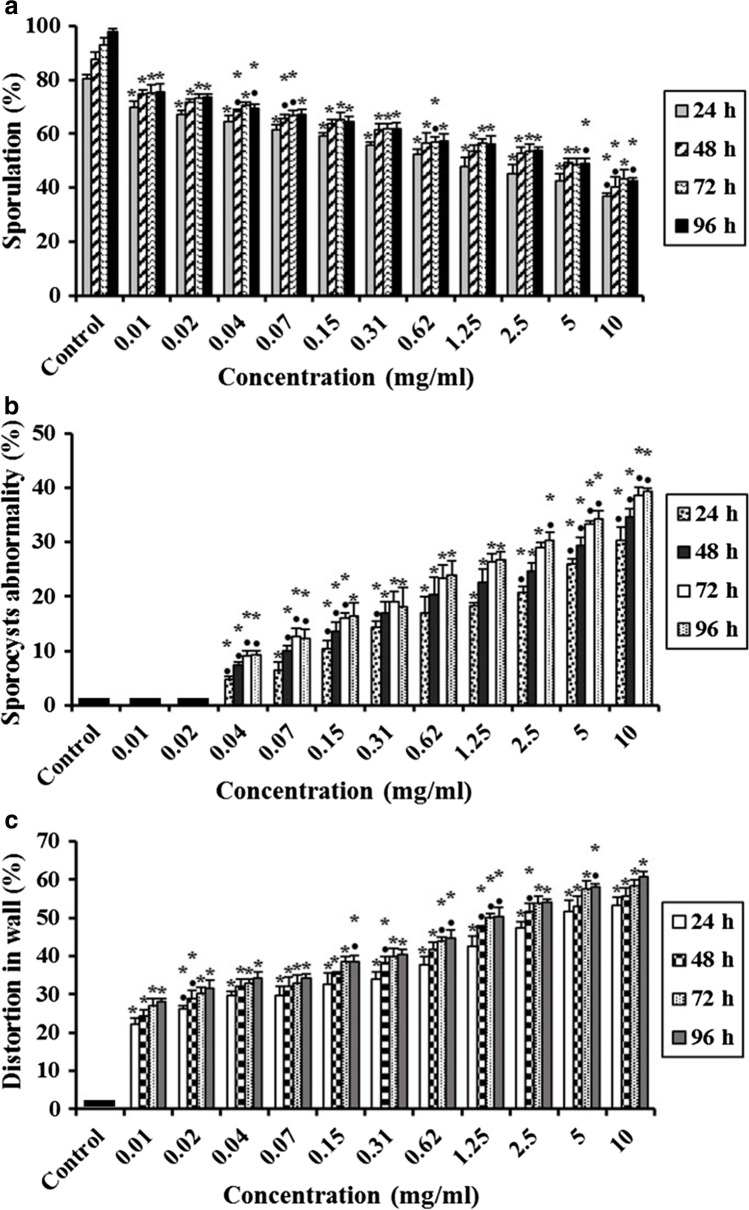
Effect of various concentrations of ultrasonicated ethanolic extract of *Rosmarinus officinalis*-chitosan based nanoparticles at pH 5 (UERRO-CsNPs5) on *E. tenella* oocysts. **a** Oocysts sporulation (%), **b** Sporocysts abnormality (%), and **c** Distortion in wall (%). Data are presented as means ± standard deviation. ^*^Significant (P ≤ 0.05) when compared to control group. ^•^Significant (P ≤ 0.05) when compared with the previous lower concentration

This study indicated that the statistical analysis of unsporulated oocysts treated with different concentrations (10, 5, 2.5, 1.25, 0.62, 0.31, 0.15, 0.07, 0.04, 0.02 and 0.01 mg/ml) of UERRO-CsNPs3 and UERRO-CsNPs5 revealed that all the tested concentrations exhibited significant decreases (P ≤ 0.05) in the sporulation (%) in a dose dependent manner related to the control group (Figs. 7a and 8a).

Concerning UERRO-CsNPs3, the lowest sporulation (%); (39.67 ± 0.58%, 43.00 ± 1.00%, 47.33 ± 1.15% and 47.00 ± 1.73% after 24, 48, 72 and 96 h, respectively) was recorded at 10 mg/ml followed by the lower concentrations reaching to the lowest concentration (0.01 mg/ml) that showed the lowest sporulation (%); 73.33 ± 2.52%, 78.67 ± 1.53%, 79.67 ± 1.15% and 78.33 ± 0.58% after 24, 48, 72 and 96 h, respectively compared to the control (Fig. 7a). The sporulation (%) in UERRO-CsNPs3 had begun to be constant after 72 h to be the same after 48 h in all the tested concentrations. In addition, all the tested concentrations except (0.02 and 0.01 mg/ml) in UERRO-CsNPs3 caused significant (P ≤ 0.05) abnormalities (%) in the sporocysts inside oocysts comparing to the control. As the concentration of UERRO-CsNPs decreased, the sporocyst abnormalities (%) decreased. Using 10 mg/ml of UERRO-CsNPs3 lead to sporocysts abnormalities with a percentage reached to 29.67 ± 1.53%, 34.33 ± 1.53%, 35.67 ± 2.08% and 38.67 ± 1.53% after 24, 48, 72 and 96 h, respectively, while the concentration (0.04 mg/ml) caused the lowest sporocysts abnormalities (%) reached to 3.33 ± 0.58%, 6.33 ± 1.53%, 8.33 ± 0.58% and 8.33 ± 1.15% after 24, 48, 72 and 96 h, respectively (Fig. 7b). Moreover, all the tested concentrations (10, 5, 2.5, 1.25, 0.62, 0.31, 0.15, 0.07, 0.04, 0.02 and 0.01 mg/ml) of UERRO-CsNPs3 demonstrated significant (P ≤ 0.05) distortion in oocysts wall (%) in a dose dependent manner. The concentration 10 mg/ml demonstrated the highest significant (P ≤ 0.05) distortion in oocysts wall with a percentage of 52.33 ± 2.52%, 54.67 ± 2.52%, 56.67 ± 1.53% and 59.33 ± 0.58% after 24, 48, 72 and 96 h, respectively, while the lowest concentration (0.01 mg/ml) had the lowest significant (P ≤ 0.05) distortion in oocyst wall (%) as 21.33 ± 1.53%, 22.33 ± 2.08%, 25.00 ± 1.00% and 25.33 ± 1.53% after 24, 48, 72 and 96 h, respectively in comparison to the control group (Fig. 7c).

On the other hand, in case of UERRO-CsNPs5, the maximum sporulation (%) (37.00 ± 1.00%, 40.33 ± 3.51%, 43.33 ± 3.21% and 42.67 ± 1.15% after 24, 48, 72 and 96 h, respectively) was seen in the highest concentration (10 mg/ml) followed by lower concentrations and the minimum sporulation (%) (70.00 ± 2.00%, 74.67 ± 1.53%, 75.00 ± 3.00% and 75.67 ± 3.06% after 24, 48, 72 and 96 h, respectively) was observed in the lowest concentration (0.01 mg/ml) comparing to the control group (Fig. 10a). In addition, the sporulation (%) in UERRO-CsNPs5 had begun to be constant after 72 h to be the same after 48 h in all the tested concentrations. This means that these nanoparticles had stopped sporulation after 72 h (Fig. 8a). Also, all the tested concentrations except (0.02 and 0.01 mg/ml) in UERRO-CsNPs5 caused significant (P ≤ 0.05) abnormalities (%) in the sporocysts inside oocysts comparing to the control. As the concentration of UERRO-CsNPs decreased, the sporocyst abnormalities (%) decreased. The highest concentration (10 mg/ml) of UERRO-CsNPs5 demonstrated the highest sporocysts abnormalities with a percentage reached to 30.33 ± 2.52%, 34.67 ± 1.53%, 38.67 ± 1.53% and 39.33 ± 0.58% after 24, 48, 72 and 96 h, respectively, while the concentration (0.04 mg/ml) revealed the lowest sporocysts abnormalities (%) reached to 4.67 ± 0.58%, 7.33 ± 0.58%, 9.00 ± 1.00% and 9.33 ± 0.58% after 24, 48, 72 and 96 h, respectively (Fig. 8b). Moreover, all the tested concentrations (10, 5, 2.5, 1.25, 0.62, 0.31, 0.15, 0.07, 0.04, 0.02 and 0.01 mg/ml) of UERRO-CsNPs5 demonstrated significant (P ≤ 0.05) distortion in oocysts wall (%) in a dose dependent manner. The highest concentration (10 mg/ml) lead to significant (P ≤ 0.05) distortion (%) in oocyst wall as 53.33 ± 2.08%, 55.67 ± 2.08%, 58.33 ± 1.53% and 60.67 ± 1.53% after 24, 48, 72 and 96 h, respectively compared to the control, while the lowest concentration (0.01 mg/ml) had the lowest significant (P ≤ 0.05) distortion in oocyst wall as 22.33 ± 1.53%, 24.33 ± 1.53%, 27.00 ± 2.00% and 28.00 ± 1.00% after 24, 48, 72 and 96 h, respectively as related to the control group (Fig. 8c).

## Light Micrographs of *E. tenella* Oocysts Treated with UEERO, Free CsNPs3, free CsNPs5, UEERO-CsNPs3 and UEERO-CsNPs5

**Fig. 9:**
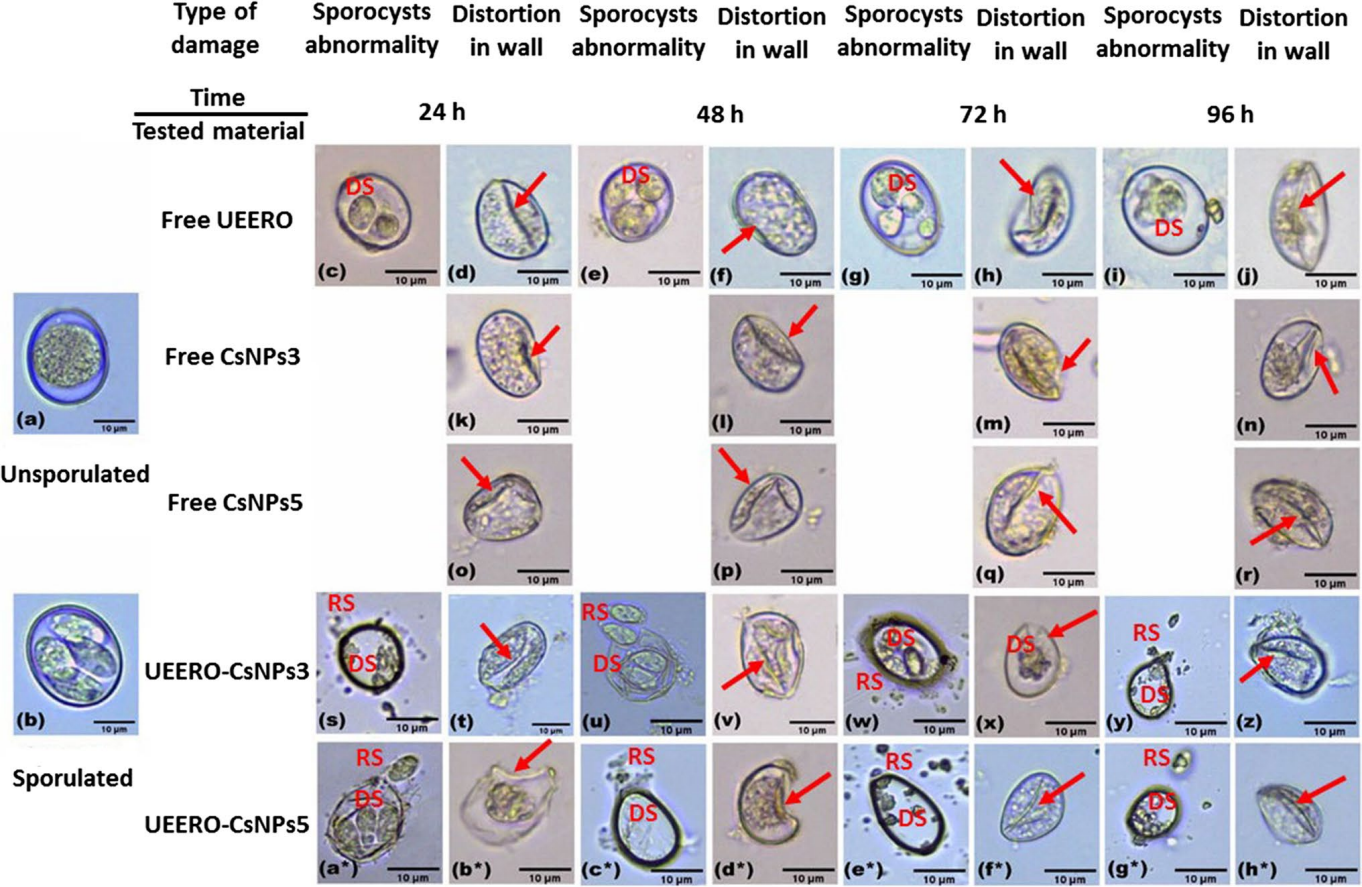
Photomicrographs of *E. tenella* oocysts treated with 10 mg/ml of UERRO free CsNPs3 free CsNPs5 UERRO-CsNPs3 and UERRO-CsNPs5. UEERO; ultrasonicated ethanolic extract of *Rosmarinus officinalis* free CsNPs3; free chitosan nanoparticles at pH 3 free CsNPs5; free chitosan nanoparticles at pH 5 UEERO-CsNPs3; ultrasonicated ethanolic extract of *Rosmarinus officinalis*-chitosan based nanoparticles at pH 3 UEERO-CsNPs5; ultrasonicated ethanolic extract of *Rosmarinus officinalis*-chitosan based nanoparticles at pH 5. Arrow; deformation in oocyst wall RS; released sporocysts DS; damaged sporocysts. Scale bar = 10 µm

The changes induced by 10 mg/ml UEERO on oocysts including damage of its inside sporocysts and deformations in its wall are shown in Fig. 9C-J. Also, 10 mg/ml of UEERO showed a significant (P ≤ 0.05) decrease in oocysts length to 13.66 ± 1.07, 14.07 ± 0.32, 14.47 ± 1.09 14.98 ± 0.75 µm and in width to 10.18 ± 1.16, 10.20 ± 1.06, 9.77 ± 2.08 and 9.49 ± 1.92 µm after 24, 48, 72 and 96 h respectively comparing to the control (Table SIII).

Additionally, 10 mg/ml of both free CsNPs3 and 5 had remarkable deformations in oocysts wall (Fig. 9k-r) relative to the control oocysts (Fig. 9a and b). Moreover, 10 mg/ml of free CsNPs3 showed a significant (P ≤ 0.05) decrease in oocysts length to 14.96 ± 1.26, 13.55 ± 0.93, 13.95 ± 1.16 and 13.06 ± 1.65 µm and in width to 9.93 ± 2.19, 9.37 ± 1.24, 9.09 ± 1.39 and 9.87 ± 1.03 µm after 24, 48, 72 and 96 h respectively in comparison to the control (Table SIII). On the other hand, 10 mg/ml of free CsNPs5 exhibited a significant (P ≤ 0.05) decrease in oocysts length by 13.06 ± 1.65, 13.75 ± 2.18, 13.68 ± 1.11 and 13.88 ± 1.34 µm and in width by 9.87 ± 1.03, 9.29 ± 1.35, 10.38 ± 1.42 and 10.13 ± 1.70 µm after 24, 48, 72 and 96 h respectively related to the control (Table SIII).

Also, 10 mg/ml of UEERO-CsNPs3 affected negatively on oocysts shape causing abnormal deformation in its wall with collapsing (Fig. 9t, v, x and z) and destruction of its inside sporocysts with observed released destructive sporocysts around the oocysts after 24, 48, 72 and 96 h (Fig. 9s, u, w and y). The same relative negative effects on oocysts were also seen using 10 mg/ml of UERRO-CsNPs5 as it caused observed abnormal changes in its wall (Fig. 9a*, c*, e* and g*) and destroying in its inside sporocysts with observed released destructive sporocysts outside the oocysts after 24, 48, 72 and 96 h (Fig. 9b*, d*, f* and h*). Moreover, 10 mg/ml of UEERO-CsNPs3 revealed a significant (P ≤ 0.05) decrease in oocysts length to 13.06 ± 4.00, 13.71 ± 3.70, 12.13 ± 2.62 and 11.60 ± 1.13 µm and in width by 9.81 ± 1.45, 9.40 ± 0.50, 8.72 ± 2.39 and 7.34 ± 1.50 µm after 24, 48, 72 and h respectively in comparison to the control (Table SIII). On the other hand, 10 mg/ml of UEERO-CsNPs5 exhibited a significant (P ≤ 0.05) decrease in oocysts length to 12.54 ± 0.54, 12.43 ± 2.01, 12.37 ± 1.30 and 10.45 ± 2.25 µm and in width to 9.59 ± 0.90, 7.14 ± 1.00, 7.76 ± 0.43 and 7.56 ± 2.35 µm after 24, 48, 72 and h respectively in comparison to the control (Table SIII).

## Field Emission Scanning Electron Micrographs (FESEM) of *E. Tenella* Oocysts Treated With UEERO, Free CsNPs3, Free CsNPs5, UEERO-CsNPs3 and UEERO-CsNPs5

**Fig. 10:**
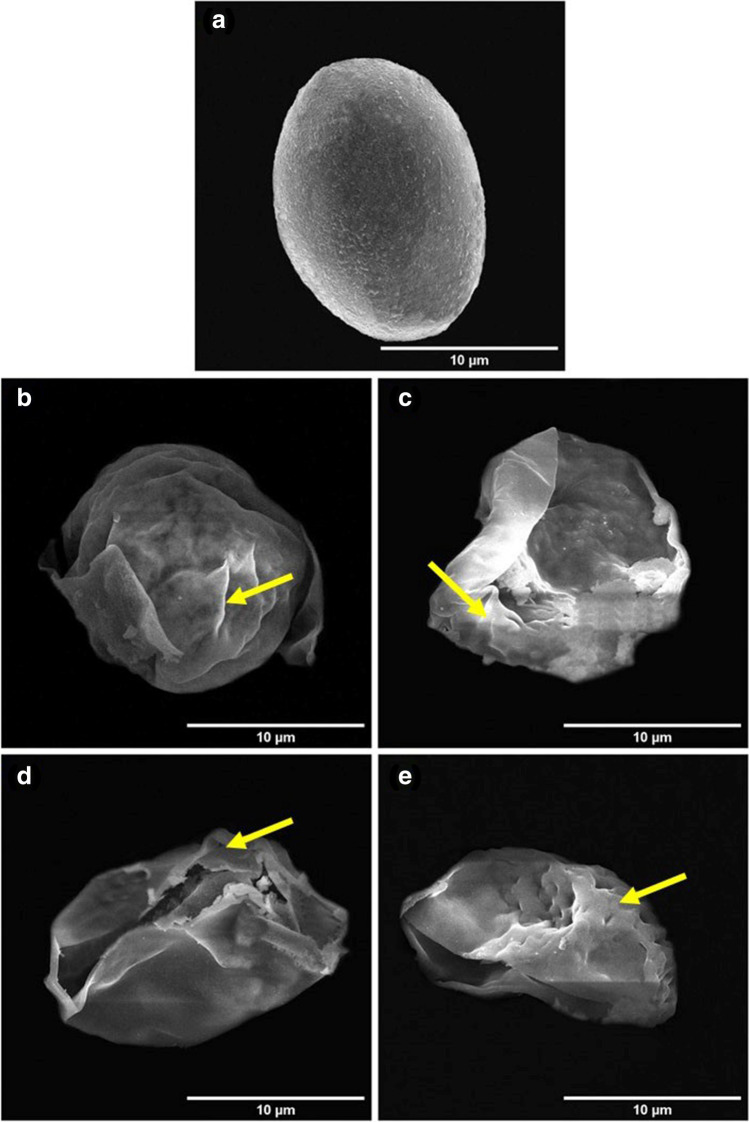
FESEM of *E. tenella* oocysts treated with 10 mg/ml of UEERO, **a** Oocysts from control medium, **b** Oocysts appeared with wrinkling (arrow) in its wall after 24 h, **c** Oocysts are likely to be exploded and opened with wrinkling (arrow) wall after 48 h, and** d**,** e** Oocysts opened with wrinkles (arrow) shrinkage and collapsing after 72 and 96 h. Scale bar = 10 µm

In addition to light micrographs, high resolution micrographs were taken using FESEM to confirm changes occur *in vitro*. FESEM indicated that oocysts incubated in 10 mg/ml UEERO appeared wrinkled after 24 h (Fig. 10b), while after 48 h, the oocysts were likely to be exploded and opened (Fig. 10c). Moreover, the oocysts appeared to be collapsed with shrinkage after 72 and 96 h (Fig. 10d and e) in comparison to oocysts from control incubations that exhibited typically consistent wall and ovoid in shape (Fig. 10a). Additionally, the concentration 10 mg/ml free CsNPs3 has a remarkable negative effect on oocysts morphology as the appeared to be wrinkled with cracks in their wall after 24 and 48 h (Fig. S4b and c) and oocysts continued to be collapsed with shrinkage and crinkles its wall after 72 and 96 h (Fig. S4d and e) in comparison to control (Fig. S4a). On the other hand, the use of 10 mg/ml free CsNPs5 affected the oocysts to be appeared with cracks its wall and wrinkling in their wall morphology from 24 to 96 h (Fig. S5b-e) related to the control oocysts (Fig. S5a). Moreover, the treated oocysts with 10 mg/ml of UEERO-CsNPs3 were shown with creases in its wall after 24 h (Fig. S6b) and are likely to be exploded and opened after 48 h (Fig. S6c), while they appeared with remarkable wrinkles and collapsing after 72 and 96 h (Fig. S6d and e) compared to the control (Fig. S6a). Additionally, using 10 mg/ml of UEERO-CsNPs5 lead to explosion and opening of oocysts after 24 h (Fig. S7b) and oocysts appeared to have creases and wrinkles in its wall after 48 h (Fig. S7c). Oocysts continued to be wrinkled with collapsing and shrinkage after 72 and 96 h (Fig. S7d and e) in comparison to the control (Fig. S7a).

## Discussion

In the present study, The HPLC analysis revealed that EERO consists of 13 different groups polyphenolic compounds including 8 phenolic acids, one phenolic aldehyde and 4 flavonoid compounds. It is evident that the highest phenolics compound in EERO was recorded for ferulic acid; 1009.25 µg/g, gallic acid; 1004.16, and ellagic acid; 739.12 µg/g, while the lowest content was recorded for coumaric acid; 72.51 µg/g and cinnamic acid; 40.81 µg/g. These results are in contrast with Ragab *et al*. (2015) [55], who reported that the cinnamic acid was the most abundant phenolic compound in rosemary leaves extracted by HPLC. Ferulic acid is considered to be a superior antioxidant and possesses many physiological functions, including anti-inflammatory, antimicrobial, anticancer, antidiabetic effects and immunostimulant properties [56]. Gallic acid and its derivatives such as propyl gallate, octyl gallate are characterized by antioxidant nature with emphasis on antimicrobial, anti-inflammatory, anticancer, cardioprotective, gastroprotective, and neuroprotective effects [57]. Additionally, in this study the highest flavonoid content was kaempferol; 259.04 µg/g followed by quercetin; 179.23 µg/g. Kaempferol and its glycosylated derivatives have been shown to be cardioprotective, neuroprotective, anti-inflammatory, antidiabetic, antioxidant, antimicrobial, antitumor, and have anticancer activities [58]. Quercetin is a flavonoid found in fruits and vegetables, has unique biological properties including anti-carcinogenic, anti-inflammatory, antiviral, antioxidant and psychostimulant activities, as well as the ability to inhibit lipid peroxidation, platelet aggregation and capillary permeability [59].

EERO was also subjected to EDX analysis to ensure the purity and elemental composition of the extract. It revealed that the purity of EERO was about 99.93% with about 0.06% of potassium impurities.

Also, CsNPs were synthesized using ionic gelation method in which the negatively charged phosphoric and OH^−^ anions are ionically crosslinked with the positively charged amino groups of Cs. pH represents a fundamental parameter that controls the aggregation behavior of CsNPs and so their shape, size and zeta potential of synthesized NPs [60].

SEM used to reveal the morphology of NPs as a fundamental property that indicates the surface features of the synthesized NPs and thus, it will influence the loading, release, pharmacokinetics and cellular/oocyst interaction [61]. The change of NPs shape occurs as it was reported that Cs solubility increases at acidic pH and the process of amino group protonation to NH_3_^+^ forming a polycation in acidic media. However, CsNPs tend to deprotonate to neutral NH_2_ group and NPs agglomeration due to physical forces formed [62]. Consequently, it affects the cross-linking strength and orientation of synthesized NPs.

Scheme 1 showed that CsNPs tend to protonate NH_2_ groups to NH_3_^+^ and have a surface positive charge at acidic pH that help in the formation of one of attraction forces with about 13 active components in UERRO extract. These attraction forces formed between macromolecules (UEERO) and small particles (CsNPs) includes electrostatic forces, van der Waals forces, hydrogen bonds and hydrophobic forces [63].

DLS technique used to investigate the particle size, homogeneity and surface charge of the synthesized NPs. In this study, the obtained size with acceptable values of polydispersity makes the synthesized NPs suitable for cellular penetration and drug/ligand delivery [64].

Zeta potential is a very important physiochemical parameter that affects the surface charge of NPs and thus, their cellular uptake and biodistribution [65]. The positive zeta potential of NPs increases the opportunity of NPs to react with most of cellular membranes due to their negative charge and thus their permeability [66]. Also, the successful interaction between UEERO and CsNPs was observed due to the decrease in in positive charge of free NPs after interaction with negatively charged UEERO particles. Generally, the charge of NPs and their formulations was considered neutral at the range between—10 to + 10 mV while those with charge less than – 30 mV and larger than + 30 mV are considered strongly anionic and cationic, respectively [67].

XRD is a technique used to investigate the expected deformation in crystal structure of synthesized samples after interaction. Thus, the UEERO peak exhibited a decrease after interaction with CsNPs until the complete disappearance at pH 5 as indication of successful loading of UEERO particles inside and on the surface of CsNPs [68]. FTIR technique depends on molecular vibrations that indicate the functional groups of synthesized NPs. It showed similar patterns as previously mentioned [52, 53]. Also, it confirmed the good interaction of UEERO and CsNPs observed in the shift of keto group and C-O bands of UEERO.

HPLC technique is used to separate, identify and quantify each component in mixtures and nano-formulations [69]. As indicated by HPLC chromatogram of this study, caffeic and rosmarinic acids in UEERO-CsNPs3 were separated at retention time of 3.41 and 11.22 min, respectively while in UEERO-CsNPs5, they were observed at retention time of 3.80 and 11.32 min, respectively. This reveals that both acids were repeatedly retained in UEERO-CsNPs3 and UEERO-CsNPs5, stating very good resolution for both acids [70]. Consequently, the total LE of UEERO particles on CsNPs5 and CsNPs3 calculated according to Espinoza *et al*. [42] using UV–vis spectrophotometer was about 80.05 and 64.39%, respectively that may be due to the enhanced positive charge of NPs.

Unsporulated oocysts of *E. tenella* in this study had showed ovoid shape with an inside zygote and surrounded by two layered oocyst wall (outer and inner), while sporulated oocysts appeared ovoid and surrounded by two layered oocyst wall (outer and inner) with 4 sporocysts, each had 2 sporozoites inside. The same as reported by Kasem *et al*. [19]. Additionally, oocysts of *E. tenella* of this study had an average size of 22.62 ± 1.35 µm in length, 18.81 ± 1.58 µm in width and had a shape index of 1.21 ± 0.11. Debbou-Iouknane *et al*. [71] reported that *E. tenella* oocysts has a length of 22.7 ± 2.4 and a width of 18.9 ± 2.9 with a shape index of 1.20. Khaier *et al*. [72] showed that *E. tenella* oocyst had measurements of 19.63 µm in length and 17.02 µm in width and the shape index of 1.16 is recorded.

Ultrasonication is a technique used to decrease size and increase dispersibility in the extract. This can be occurred as ultrasonic waves produce bubbles and cavities between particles in solutions breaking the weak van der Waals and other attraction forces that cause intra-molecules cleavage and thus, reducing the particles’ size that will enhance the absorption of UEERO inside the oocysts [73]. Consequently, the present study revealed that UEERO had an inhibitory anticoccidial effect on sporulation (%) of *E. tenella* oocysts in a dose dependent manner as compared to control group. The highest concentration (10 mg/ml) of UERRO showed the lowest sporulation percentage. Whereas, the lowest concentration (0.01 mg/ml) of UERRO showed the highest sporulation (%) comparing to the control group. This corresponds to that the highest concentration (10 mg/ml) revealed the highest sporulation inhibition (%) compared to the other concentrations. This agrees with Molan *et al*. [74] who reported that *Psidium guajava* extract could reduce the sporulation percentage by inhibiting or inactivating the enzymes responsible for the sporulation process. In another study, Abbas *et al*. [75] mentioned that *Vitis venifera* extract had an inhibitory effect on sporulation (%) of *Eimeria* oocysts in a dose dependent manner as compared to control groups. This is also in accordance with the findings of Ishaq *et al*. [18] who showed that ethanolic leaf extract of *Citrus aurantium* possess *in vitro* anticoccidial effect against the unsporulated oocysts of *E. tenella* in a concentration dependent manner. Several investigators found that rosemary bioactive properties are connected with the presence of phenolic compounds, especially flavonoids and diterpenes, such as carnosic acid and carnosol that characterized with its antioxidant activity [76–78]. These researchers further reported that extracts containing polyphenolic compounds may have the ability to inhibit enzymes responsible for the sporulation process of the coccidian oocysts [79]. Additionally, in the current study, the highest concentration (10 mg/ml) of UEERO showed the highest percentage in sporocysts abnormalities with the highest distortion (%) in oocyst wall, while the lowest concentration (0.01 mg/ml) exhibited the lowest sporocysts abnormalities (%) with the lowest distortion (%) in oocyst wall. Abbas *et al*. [75] reported that *Vitis venifera* extract caused a damage of *Eimeria* oocysts in a dose dependent manner as compared to control groups. Jones *et al*. [80] suggested that extracts may penetrate the cell wall of oocysts and cause a loss of intracellular components. Also, Cedric *et al*. [81] explained that *Psidium guajava* extracts might have penetrated the wall of the oocysts and damaged the cytoplasm (sporocysts).

In the present study, concerning free CsNPs3 and free CsNPs5, the lowest sporulation (%) was observed at 10 mg/ml causing significant (P ≤ 0.05) distortion in the oocyst wall (%), while the lowest concentration (0.01 mg/ml) showed non-significant changes in the sporulation percentage comparing to the control but it caused significant (P ≤ 0.05) distortion in the oocyst wall (%). In Egypt, CsNPs have recently been used as anti-bacterial [82, 83] and anti-protozoal agents (anti-Giardia and anti-Toxoplasma) [84–86] with effective results. Elmi *et al*. [37] demonstrated that nano-chitosan could be used as an anti-parasitic nano-compound against *Plasmodium falciparum, Giardia lamblia* and *Trichomonas vaginalis*. The destructive mechanism of CsNPs might be due to its small size. The small size of the nanoforms usually expose large surface area to volume ratio [87] and increase the *in vitro* efficacy by increasing the dissolution and bioavailability. The destructive effect could therefore potentially increase the electrostatic interaction between CsNPs and oocysts [32].

Additionally, concerning UEERO-CsNPs3 and UERRO-CsNPs5, the lowest sporulation (%) was recorded at 10 mg/ml followed by the lower concentrations reaching to the lowest concentration (0.01 mg/ml) that showed the lowest sporulation (%) compared to the control. The sporulation (%) in UERRO-CsNPs3 and UERRO-CsNPs5 had begun to be constant after 72 h to be the same after 48 h in all the tested concentrations. Using 10 mg/ml of UERRO-CsNPs3 and UERRO-CsNPs5 led to the highest sporocysts abnormalities and demonstrated the highest significant (P ≤ 0.05) distortion in oocysts wall, while the concentration (0.04 mg/ml) caused the lowest percentage in sporocysts abnormalities related to the control. Moreover, the lowest concentration (0.01 mg/ml) in both UERRO-CsNPs3 and UERRO-CsNPs5 had the lowest significant (P ≤ 0.05) distortion in oocyst wall (%) in comparison to the control group. Bell *et al*. [87] and Barhoum *et al*. [88] indicated that the conjugation of *Commiphora molmol* into the chitosan nanofibers permitted the adsorption of *C. molmol* to the surface of the wall of the oocysts, contributing to a shift in its integrity and permeability leading to their damage [32].

Light micrographs of *E. tenella* oocysts treated with UEERO, free CsNPs3, free CsNPs5, UEERO-CsNPs3 and UEERO-CsNPs5 are also taken in this study. The concentration 10 mg/ml UEERO on oocysts induced damage of its inside sporocysts and deformations in its wall. Also, 10 mg/ml of UEERO showed a significant (P ≤ 0.05) decrease in oocysts length and width comparing to the control. These results are in agreement with Abbas *et al*. [3] who demonstrated that *Trachyspermum ammi* extract damaged the morphology of oocysts in terms of shape, size and number of sporocysts. The plant *R. officinalis* is a rich source of polyphenolic compounds and is the basis of widely commercialized plant extracts known for their potent antioxidant activity [89, 90] Arlette *et al*. [91] indicated that natural polyphenolic components from medicinal plants have been reported to inhibit cell invasion of *E. tenella* sporozoites *in vitro*.

## Conclusions

It could be concluded that the ethanolic extract of *Rosmarinus officinalis* and its chitosan-based nanoparticles had the potential to be used as an *in vitro* anticoccidial agents against *E. tenella* oocysts of chickens. Further studies are required to study the possible adverse effects of ethanolic extract of *Rosmarinus officinalis* and its chitosan-based nanoparticles and to prove their anticoccidial abilities *in vivo.*

## Supplementary Information

Below is the link to the electronic supplementary material.

Supplementary file1 (DOCX 988 KB)
