# Supplementary material for: In Vitro Study to Evaluate the Efficacy of Ultrasonicated Ethanolic Extract of Rosmarinus officinalis and its Chitosan-Based Nanoparticles Against Eimeria tenella Oocysts of Chickens
Source: AAPS PharmSciTech. 2022 Nov 3;23(8):295. doi: 10.1208/s12249-022-02445-z (PMC9633124; doi:10.1208/s12249-022-02445-z)
Supplement: Supplementary file 1 — Supplementary file1 (DOCX 988 KB) [file 12249_2022_2445_MOESM1_ESM.docx]

**
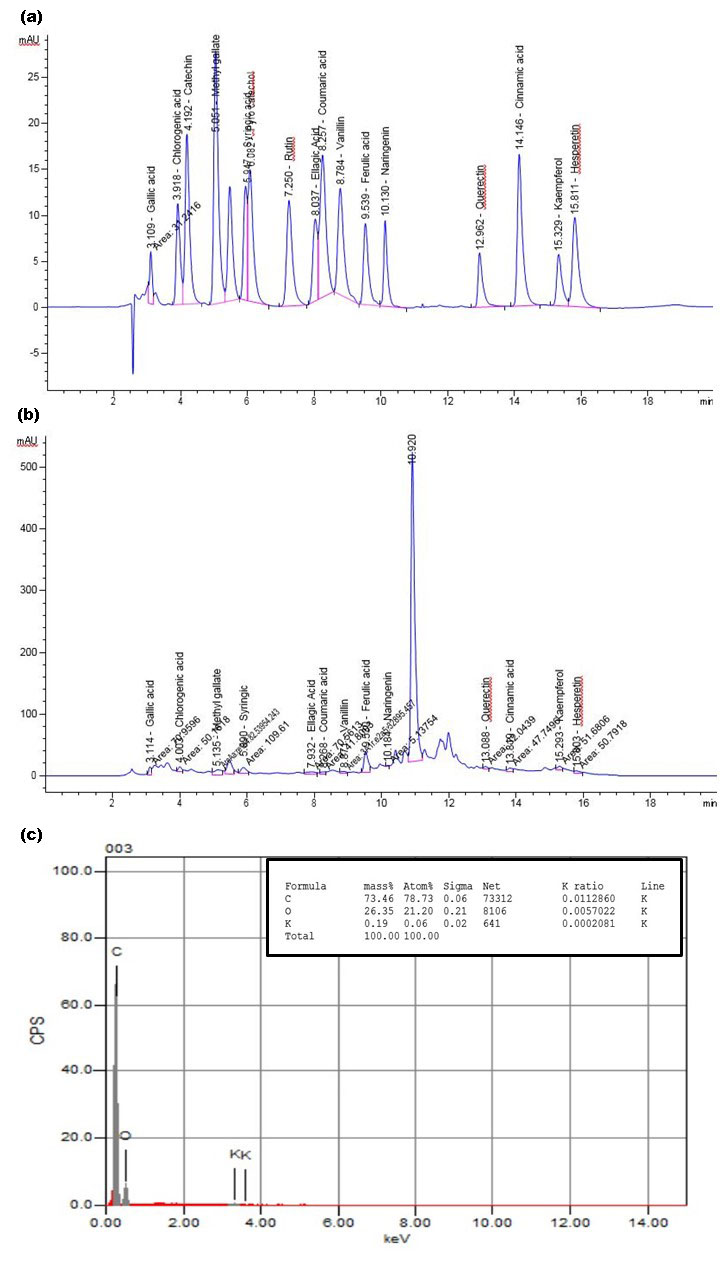
**

**Figure S1** HPLC chromatogram of multi-standards **(a)**, EERO **(b**), and EDX analysis of EERO **(c)**.

*
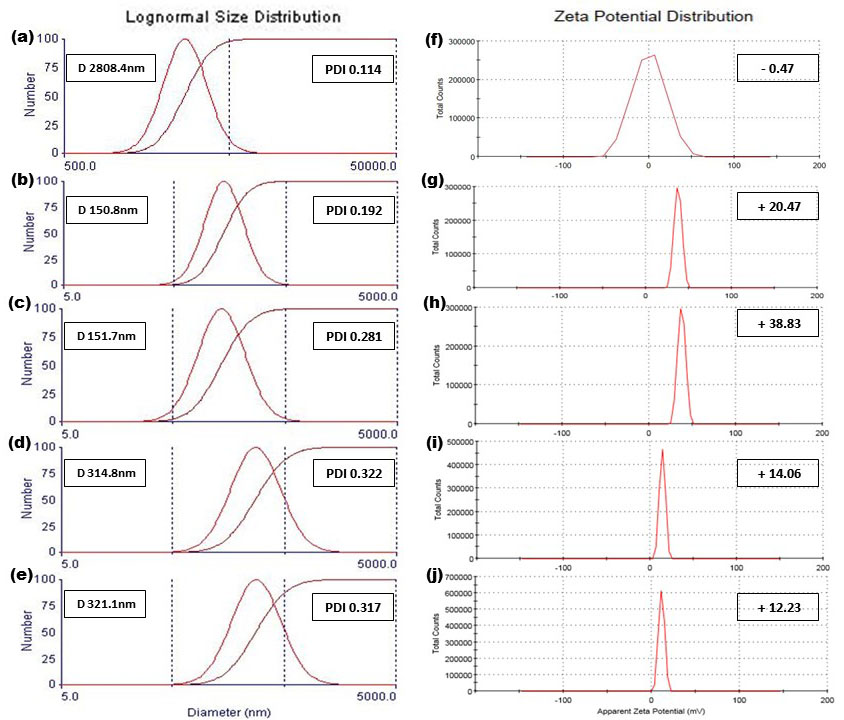
*

**Figure S2** Hydrodynamic size, polydispersity index (PDI) of free UEERO **(a)**, free CsNPs3 **(b)**, free CsNPs5 **(c)**, UEERO-CsNPs3 **(d)**, and UEERO-CsNPs5 **(e)**. Zeta potential of free UEERO **(f)**, free CsNPs3 **(g)**, free CsNPs5 **(h)**, UEERO-CsNPs3 **(i)**, and UEERO-CsNPs5 **(j)**. UEERO; ultrasonicated ethanolic extract of Rosmarinus officinalis free CsNPs3; free chitosan nanoparticles at pH 3 free CsNPs5; free chitosan nanoparticles at pH 5 UEERO-CsNPs3; ultrasonicated ethanolic extract of Rosmarinus officinalis-chitosan based nanoparticles at pH 3 UEERO-CsNPs5; ultrasonicated ethanolic extract of Rosmarinus officinalis-chitosan based nanoparticles at pH 5

**
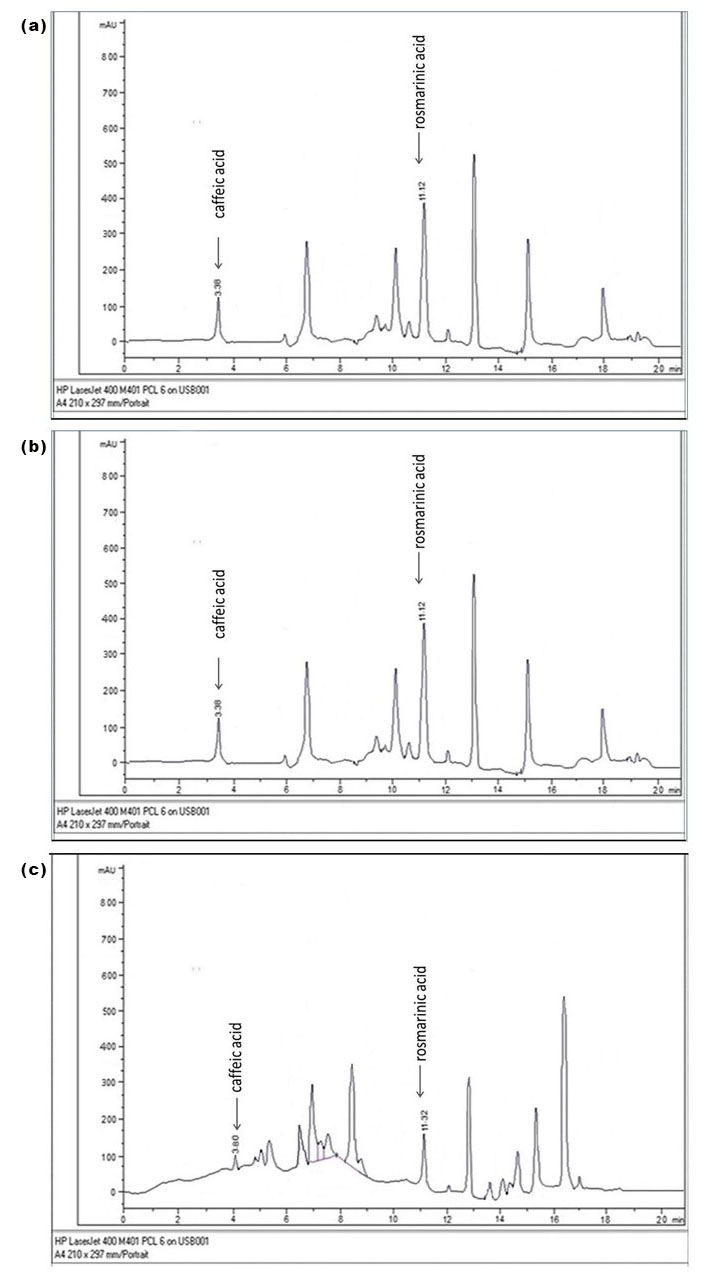
**

**Figure S3** HPLC chromatograms showing characteristic peaks of caffeic acid and rosmarinic acid in **(a)** free UEERO, **(b)** UEERO-CsNPs3, and **(c)** UEERO-CsNPs5 at their respective retention times

**
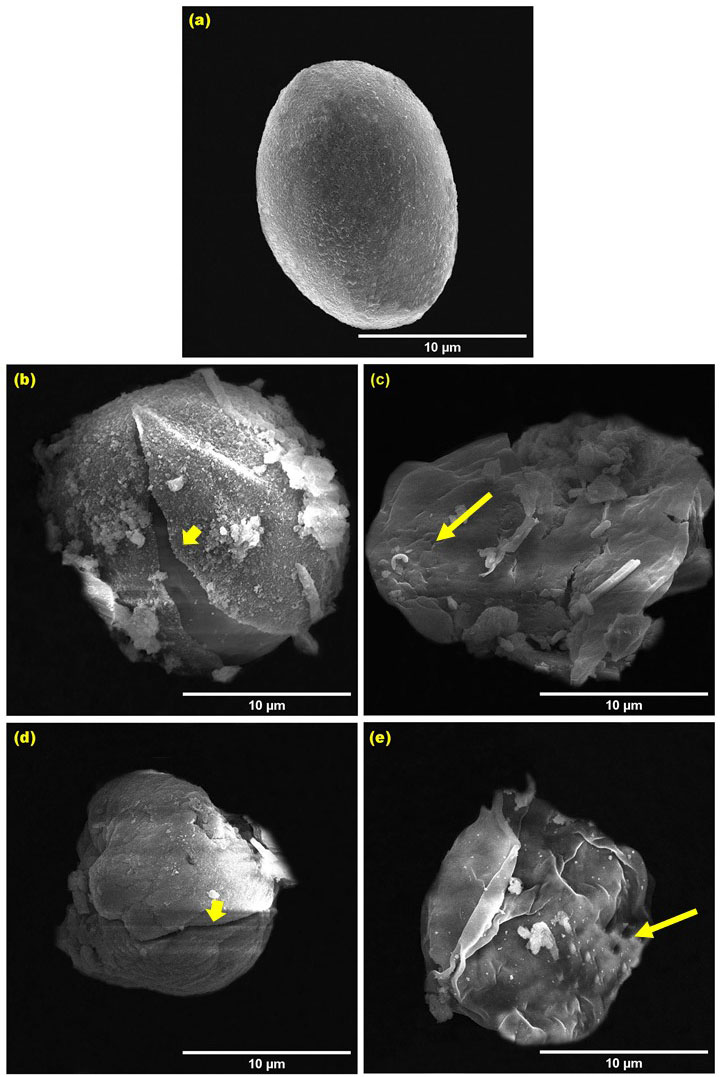
**

**Figure S4** FESEM of *E. tenella* oocysts treated with 10 mg/ml of free CsNPs3. **(a)** Oocysts from control medium, **(b)** Oocysts appeared with cracks in its wall (arrowhead) after 24 h, **(c)** Oocysts are showed to be wrinkled (arrow) and collapsed after 48 h, and **(d, e)** Oocysts continued to be collapsed with shrinkage crinkles (arrowhead) and wrinkles (arrow) and in its wall after 72 and 96 h. Scale bar=10µm

**
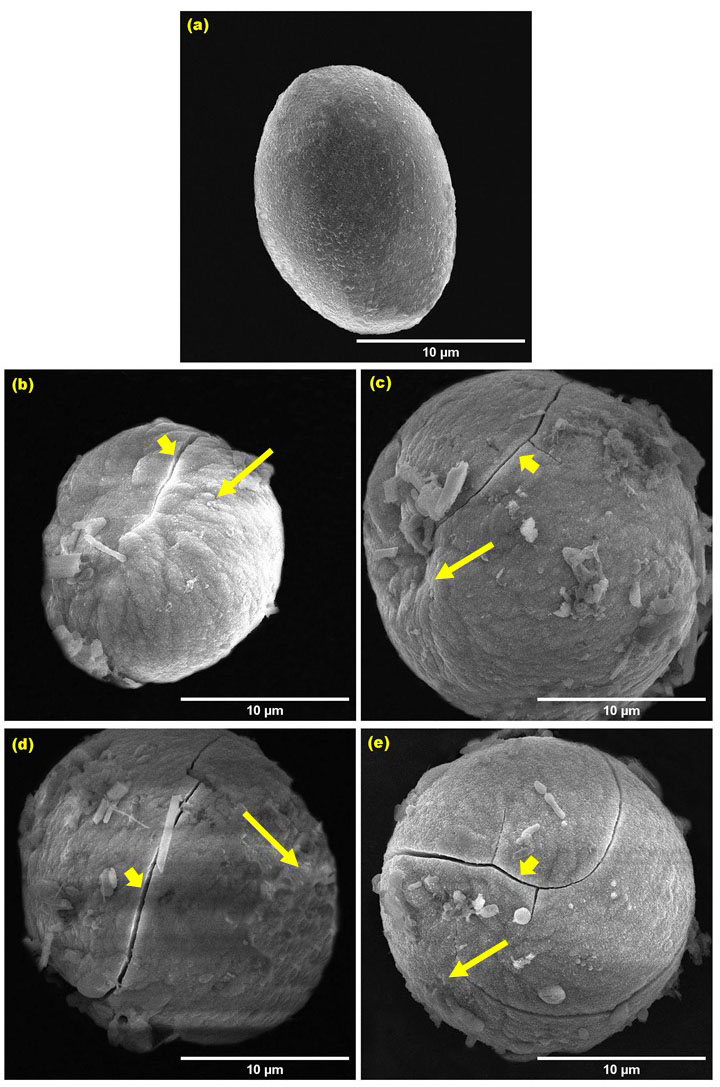
**

**Figure S5** FESEM of *E. tenella* oocysts treated with 10 mg/ml of free CsNPs5. **(a)** Oocysts from control medium, and **(b-e)** Oocysts appeared with cracks its wall (arrow) and wrinkling (arrowhead) in their wall morphology from 24 to 96 h. Scale bar=10µm

**
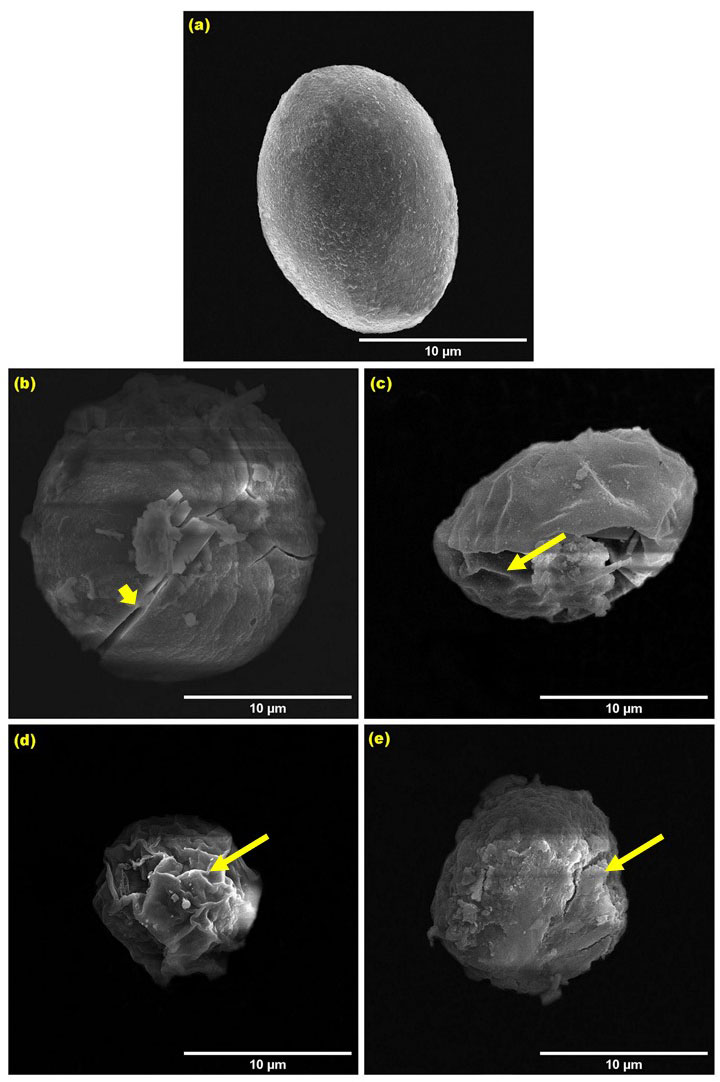
**

**Figure S6** FESEM of *E. tenella* oocysts treated with 10 mg/ml of UEERO-CsNPs3. **(a)** Oocysts from control medium, **(b)** Oocysts had creases in its wall (arrowhead) after 24 h, **(c)** Oocysts are likely to be exploded and opened with wrinkles (arrow) after 48 h, and **(d, e)** Oocysts with remarkable wrinkles (arrow) and collapsing after 72 and 96 h. Scale bar=10µm.

**
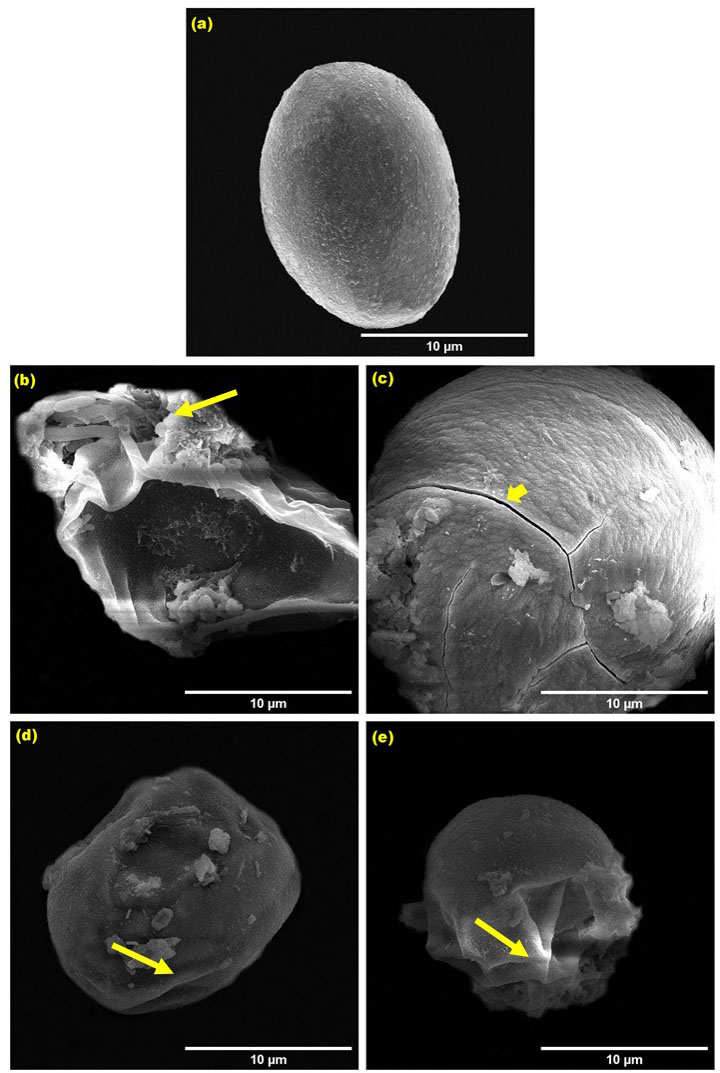
**

**Figure S7** FESEM of *E. tenella* oocysts treated with 10 mg/ml of UEERO-CsNPs5. **(a)** Oocysts from control medium, **(b)** Oocysts appeared to be exploded and opened with wrinkles (arrow) after 24 h, **(c)** Oocysts had creases in its wall (arrowhead) and wrinkles (arrow) after 48 h, and **(d, e)** Oocysts continued to be wrinkled (arrow) with collapsing and shrinkage of oocysts after 72 and 96 h. Scale bar=10µm

**Table SI** Phenolic compounds concentrations present in EERO.

| **Phenolic compound** | **Standarad** | | **EERO** | | **Chemical structure** |
| --- | --- | --- | --- | --- | --- |
|  | **Concentration (µg/ml)** | **Retention time**  **(min)** | **Concentration (µg/g)** | **Retention time**  **(min)** |  |
| Gallic acid | 8.4 | 3.109 | 1004.16 | 3.114 | C_7_H_6_O_5_ |
| Chlorogenic acid | 14 | 3.918 | 399.66 | 4.003 | C_16_H_18_O_9_ |
| Catechin | 33.75 | 4.192 | 0.00 | 4.192 | C_15_H_14_O_6_ |
| Methyl gallate | 5.1 | 5.051 | 150.54 | 5.135 | C_8_H_8_O_5_ |
| Syringic acid | 8.6 | 5.947 | 546.17 | 5.890 | C_9_H_10_O_5_ |
| Pyro catechol | 14.6 | 6.082 | 0.00 | 6.082 | C_6_H_6_O_2_ |
| Rutin | 30.5 | 7.250 | 0.00 | 7.250 | C_27_H_30_O_16_ |
| Ellagic acid | 17.15 | 8.037 | 739.12 | 7.932 | C_14_H_6_O_8_ |
| Coumaric acid | 6.6 | 8.257 | 72.51 | 8.268 | C_9_H_8_O_3_ |
| Vanillin | 6.45 | 8.784 | 72.23 | 8.871 | C_8_H_8_O_3_ |
| Ferulic acid | 6.2 | 9.539 | 1009.25 | 9.530 | C_10_H_10_O_4_ |
| Naringenin | 7.5 | 10.130 | 27.65 | 10.184 | C_15_H_12_O_5_ |
| Quercetin | 6.4 | 12.962 | 179.23 | 13.088 | C_15_H_10_O_7_ |
| Cinnamic acid | 2.9 | 14.146 | 40.81 | 13.819 | C_9_H_8_O_2_ |
| Kaempferol | 6 | 15.329 | 259.04 | 15.293 | C_15_H_10_O_6_ |
| Hesperetin | 6.6 | 15.811 | 135.50 | 15.803 | C_16_H_14_O_6_ |

**Table SII** The loading efficiency (LE) of UEERO-CsNPs and caffeic / rosmarinic acids

|  | **UEERO-CsNPs3** | **UEERO-CsNPs5** |
| --- | --- | --- |
| **Total LE** | 64.39 % | 80.05 % |
| **Caffeic acid LE** | 59.21 % | 38.28 % |
| **Rosmarinic acid LE** | 60.17 % | 31.75 % |

UEERO-CsNPs3; ultrasonicated ethanolic extract of *Rosmarinus officinalis*-chitosan based nanoparticles at pH 3 UEERO-CsNPs5; ultrasonicated ethanolic extract of *Rosmarinus officinalis*-chitosan based nanoparticles at pH 5

**Table SIII** Size of *E. tenella* oocysts treated with 10 mg/ml of UERRO, free CsNPs3, free CsNPs5, UERRO-CsNPs3 and UERRO-CsNPs5 (Data are means ± standard deviation)

| Control | Length (µm) | 22.62±1.35 | | | |
| --- | --- | --- | --- | --- | --- |
|  | Width (µm) | 18.81±1.58 | | | |
| Tested material | | Incubation time | | | |
|  |  | 24 h | 48 h | 72 h | 96 h |
| Free UEERO | Length (µm) | 13.66 ± 1.07^*^ | 14.07 ± 0.32^*^ | 14.47 ± 1.09^*^ | 14.98 ± 0.75^*^ |
|  | Width (µm) | 10.18 ± 1.16^*^ | 10.20 ± 1.06^*^ | 9.77 ± 2.08^*^ | 9.49 ± 1.92^*^ |
| Free CsNPs3 | Length (µm) | 14.96 ± 1.26^*^ | 13.55 ± 0.93^*^ | 13.95 ± 1.16^*^ | 13.06 ± 1.65^*^ |
|  | Width (µm) | 9.93 ± 2.19^*^ | 9.37 ± 1.24^*^ | 9.09 ± 1.39^*^ | 9.87 ± 1.03^*^ |
| Free CsNPs5 | Length (µm) | 13.06 ± 1.65^*^ | 13.75 ± 2.18^*^ | 13.68 ± 1.11^*^ | 13.88 ± 1.34^*^ |
|  | Width (µm) | 9.87 ± 1.03^*^ | 9.29 ± 1.35^*^ | 10.38 ± 1.42^*^ | 10.13 ± 1.70^*^ |
| UERRO-CsNPs3 | Length (µm) | 13.06 ± 4.00^*^ | 13.71 ± 3.70^*^ | 12.13 ± 2.62^*^ | 11.60 ± 1.13^*^ |
|  | Width (µm) | 9.81 ± 1.45^*^ | 9.40 ± 0.50^*^ | 8.72 ± 2.39^*^ | 7.34 ± 1.50^*^ |
| UERRO-CsNPs5 | Length (µm) | 12.54 ± 0.54^*^ | 12.43 ± 2.01^*^ | 12.37 ± 1.30^*^ | 10.45 ± 2.25^*^ |
|  | Width (µm) | 9.59 ± 0.90^*^ | 7.14 ± 1.00^*^ | 7.76 ± 0.43^*^ | 7.56 ± 2.35^*^ |

UEERO; ultrasonicated ethanolic extract of *Rosmarinus officinalis* free CsNPs3; free chitosan nanoparticles at pH 3 free CsNPs5; free chitosan nanoparticles at pH 5 UEERO-CsNPs3; ultrasonicated ethanolic extract of *Rosmarinus officinalis*-chitosan based nanoparticles at pH 3 UEERO-CsNPs5; ultrasonicated ethanolic extract of *Rosmarinus officinalis*-chitosan based nanoparticles at pH 5. ^*^Significant (P ≤ 0.05), when compared to control
